# Adaptation of visual responses in degenerating *rd10* and healthy mouse retinas during ongoing electrical stimulation

**DOI:** 10.3389/fnins.2026.1730445

**Published:** 2026-03-25

**Authors:** Archana Jalligampala, Hamed Shabani, Eberhart Zrenner, Daniel L. Rathbun, Zohreh Hosseinzadeh

**Affiliations:** 1 Department of Ophthalmology and Visual Sciences, University of Louisville, Louisville, KY, United States; 2 Theoretical Systems Neuroscience, Bernstein Center Freiburg, University of Freiburg, Freiburg, Germany; 3 Institute for Ophthalmic Research, Eberhard Karls University, Tübingen, Germany; 4 Werner Reichardt Centre for Integrative Neuroscience [CIN], Tübingen, Germany; 5 Graduate Training Center of Neuroscience/International Max Planck Research School, Tübingen, Germany; 6 Department of Ophthalmology, Detroit Institute of Ophthalmology, Henry Ford Health, Detroit, MI, United States; 7 Department of Ophthalmology, Radboud University Medical Center, Nijmegen, GA, Netherlands

**Keywords:** duration, electrical stimulation, latency, MEA, ON/OFF index, response changes

## Abstract

**Objective:**

Visual adaptation is a physiological and perceptual process by which the visual system adjusts to changes in the environment or visual stimuli. This process is fundamental to how we perceive the world around us and allows our visual system to efficiently encode and process visual information. The retina incorporates adaptation within its dozens of functionally distinct retinal ganglion cell types. Meanwhile, the field of retinal prostheses is increasing its understanding of electrical adaptation and cell-specific stimulation. However, very little is known about the interaction of visual and electrical stimulation on the adaptation of retinal ganglion cell types.

**Methods/Approach:**

Recording with a microelectrode array (MEA), we presented an ON and OFF full-field, visual stimulus to characterize various visual response parameters in healthy and degenerating *rd10* mouse retinas. We then evaluated visual response changes before and after blocks of monophasic voltage-controlled electrical pulse stimulation.

**Main results:**

A history of electrical stimulation strengthened visual responses in WT retina, even when changes attributable to *in vitro* visual adaptation were considered. In *rd10* retinas, electrical adaptation counteracted the baseline *in vitro* visual adaptation. In all cases, adaptation often affected the on and off visual response components differentially. Consequently, the ON/OFF classification of individual cells changed because of adaptation.

**Significance:**

Electrical stimulation-induced changes in the retina should be considered in the encoding of visual stimuli by retinal prosthetic devices. *In vitro* investigations for bionic vision should strive to probe electrical responsiveness after adaptation to ongoing electrical stimulation has achieved a steady state.

## Introduction

The human visual system is a remarkable entity, capable of perceiving and adapting to a range of visual stimuli, from the dim glow of starlight to the brilliance of a sunny day (Webster, 2015). Over the past century, many luminaries like Ramón y Cajal, Hartline, Barlow, Kuffler, Lettvin, Hubel, and Wiesel have collectively contributed to today’s understanding of information processing via the visual system (Kuffler, 1953; Rodieck and Stone, 1965; Hubel and Wiesel, 1998; Hartline, 1938; Barlow, 1953; Lettvin et al., 1959). Central to the remarkable dynamism of the visual system is the retina’s capacity for adaptation—to recalibrate and fine-tune its responses to suit the ever-changing demands of the visual input. There is an ever-growing body of literature illuminating the various visual adaptations occurring in the retina (Baccus and Meister, 2002; Demb, 2002; Moore et al., 2011; Tikidji-Hamburyan et al., 2015; Tikidji-Hamburyan et al., 2017; Borghuis et al., 2018).

The development of retinal prosthetic devices offers a promising solution to the vision loss experienced by individuals with retinal degenerative diseases such as retinitis pigmentosa and age-related macular degeneration (Dagnelie, 2012; Ayton et al., 2020). However, the concept of retinal adaptation takes on profound significance in the context of retinal prosthetics where adaptation may be a barrier to ongoing perception (Fernandez, 2018). Many studies have also investigated electrical adaptation in the retina (Fried et al., 2006; Jensen and Rizzo, 2007; Ray, 2010; Freeman and Fried, 2011; Lorach et al., 2015; Im and Fried, 2016). Our own lab has previously demonstrated that voltage tuning curves displayed substantial hysteresis due to adaptation to electrical stimulation occurring over many seconds (Hosseinzadeh et al., 2018). Despite these efforts, little is known about the complex interaction between electrical and visual adaptation.

A recent study in mouse retina by Baden et al. (2016) has shown that, beyond the simple on, off, sustained, and transient response types, there are at least 30 different physiological types of retinal outputs. Other recent investigations into bionic vision as a cure for blindness have additionally shown that electrical input filters can vary according to retinal ganglion cell (RGC) type, suggesting the possibility for cell-specific stimulation (Sekhar et al., 2017; Ho et al., 2018). In more recent effort, we sought to compare the differences in preferred electrical input with this well-established catalog of dozens of RGC types, relying on their functional responses to light stimulation as a reference point (Shabani et al., 2021; Shabani et al., 2024). The present study provides insights into the adaptation of on and off responses that occur when retinas receive electrical stimulation. Understanding such adaptation is an essential aspect of maximizing the effectiveness of bionic vision devices and improving the perceptual experience of recipient patients.

In this study, we used a full-field flashing visual stimulus to characterize mouse retinal ganglion cell visual responses. We tested the hypothesis that ongoing electrical stimulation alters visual responses in healthy and degenerating *rd10* mouse retinas. We further characterized the nature of these alterations and compared such adaptation to a pair of control conditions. We used an internal control condition to test whether the observed adaptation is specific to nearby, electrically responsive RGCs. We also tested whether the observed adaptation was specific to electrical stimulation or merely a byproduct of experimental and light probe conditions. The results enhance our understanding of adaptive mechanisms and electrical stimulation of the retina.

## Materials and methods

A detailed treatment of our experimental methods can be found in Rathbun et al. (2024).

### Animals

The animals were housed under standard 12 h light/12 h dark cycles with free and ample access to food and water. Adult wild-type C57Bl/6 J (Jackson Laboratory, Bar Harbor, ME, USA) and *rd10* (on a C57Bl/6 J background; Jackson Laboratory) strains were used, with age ranging from postnatal day 28 to 37 for both strains. The *rd10* strain is a well-established model for retinal degeneration in which the retina is unhealthy, but not yet blind at the ages examined. For each strain, three male and two female mice were used. For the external control condition, age-matched mice were used for both strains. For each strain of the control mice, two male mice were used. All procedures were approved by the Tübingen University committee on animal protection (Einrichtung für Tierschutz, Tierärztlichen Dienst und Labortierkunde directed by Dr. Franz Iglauer) and performed by the Association for Research in Vision and Ophthalmology (ARVO) statement for the use of animals in ophthalmic and visual research.

### Retinal preparation

For dissecting the retina, the mice were anesthetized by CO_2_ inhalation. Following CO_2_ inhalation, the mice were checked for absence of withdrawal reflex by pinching the between-toe tissue and then euthanized by cervical dislocation. Under normal room lighting, the eyes were removed to carbogenated (95% O_2_ and 5% CO_2_) artificial cerebrospinal fluid (ACSF) solution containing the following (in mM): 125 NaCl, 3.5 KCl, 2 CaCl_2_, 1 MgCl_2_, 1.25 NaH_2_PO_4_, 25 NaHCO_3_, and 25 Glucose, pH 7.4.

The cornea, lens, vitreous, and ora serrata were removed, and the retina was detached from the pigment epithelium, and the optic nerve was cut at the base of the retina. Special care was taken to remove all traces of vitreous material from the inner surface of the retina to optimize contact between the nerve fiber layer and recording electrodes. The retinas were maintained in carbogenated ACSF until needed. For recording, a retinal half was mounted ganglion cell layer down on a planar microelectrode array (MEA). Two small paint brushes were used to orient and flatten the retinal half without risking damage to the MEA. A dialysis membrane (Cellu Sep, Membrane Filtration Products Inc., Seguin, Texas, USA) mounted on a custom Teflon ring was lowered onto the retina to press it into closer contact with the MEA (Meister et al., 1994). After securing the MEA under the preamplifier, the retina was continuously superfused with carbogenated ACSF (~6 mL/min) maintained at 33^o^ C using both a heating plate and a heated perfusion cannula (HE-Inv-8 & PH01; Multi Channel Systems, Reutlingen, Germany) and stabilized for > 30 min prior to recording.

### Microelectrode Array (MEA) and data acquisition

For recording the spiking responses from the RGCs, a planar MEA containing 59 circular titanium nitride electrodes (diameter: 30 μm, interelectrode spacing: 200 μm; Multi Channel Systems, Reutlingen, Germany) arrayed in a rectilinear grid. Electrode impedance was 200–250 kΩ at 1 kHz measured using a nanoZ impedance meter (Plexon Inc., TX, USA). The MEA60 system (MCS, Reutlingen, Germany) acquired data at 50 kHz/channel with a 1 Hz–3 kHz filter and 1,100 × amplification. Defective electrodes were grounded, and electrical stimulation waveforms assigned via MEA_Select software. The raw data were recorded with MC_Rack at a rate of 50 kHz/channel with a filter bandwidth ranging from 1 Hz–3 kHz and amplification gain of 1,100.

### Electrical stimulation

**Figure 1:**
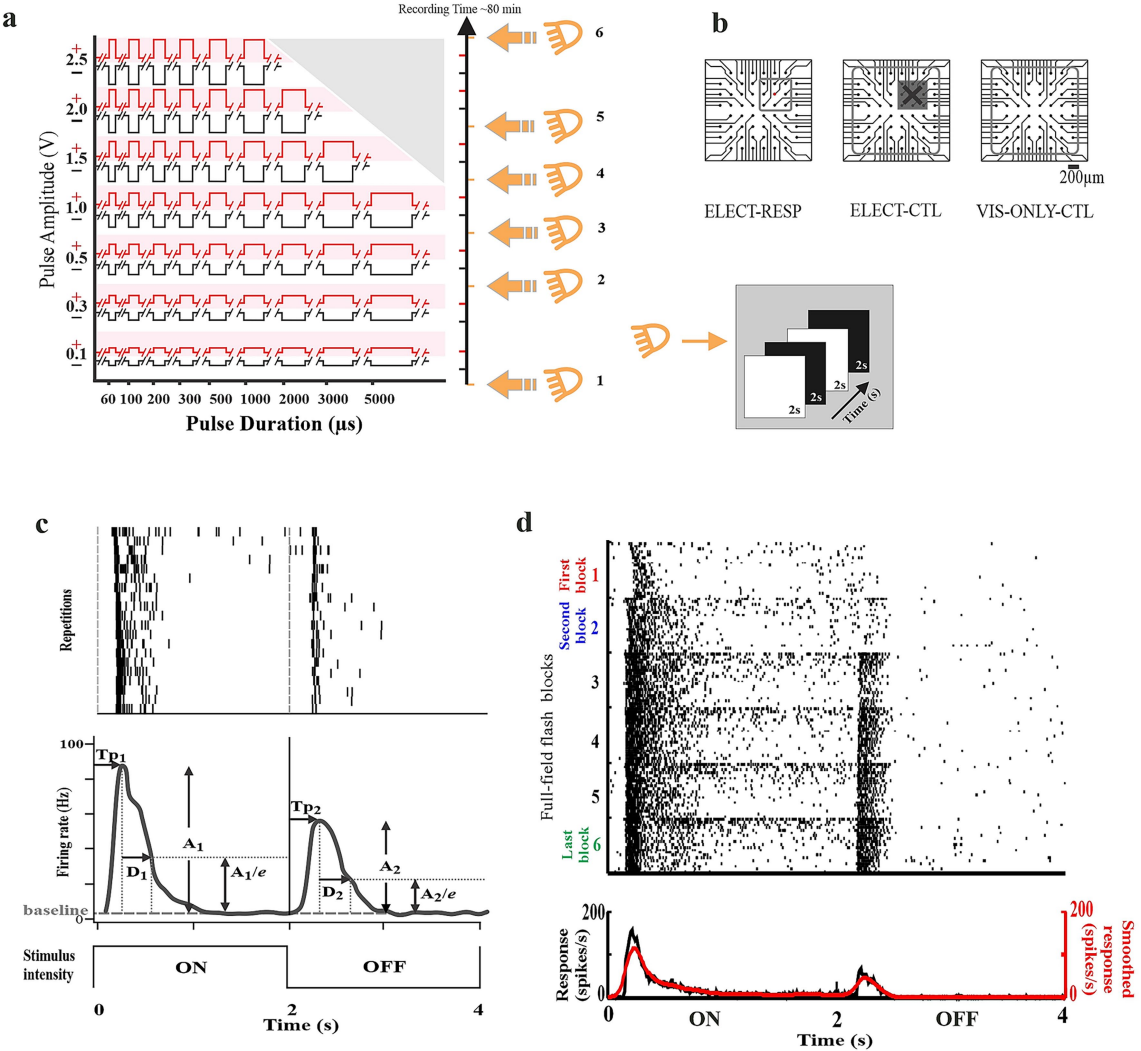
Experimental design. **(a)** Electrical stimuli were presented over a range of voltage/duration combinations. Constant-voltage stimulus blocks consisted of 5 repetitions of 9 durations. Within each repetition, the durations were randomized, 5 s separated pulses. Voltage blocks were presented from lowest to highest amplitude. Subsequent voltage blocks were separated by 150 s or greater. A block of full-field flash stimulus (20 repetitions of 2 s on and 2 s off) was interleaved between voltage blocks throughout the experiment. Voltage-duration combinations that exceeded safety limits were omitted (grey triangle). **(b)** The different test (ELECT-RESP) and control conditions (internal ELECT-CTL and external VIS-ONLY-CTL). Red denotes stimulating electrode. Open grey box indicates electrodes on which included cells were recorded. Grey box with cross indicates that cells recorded on these 9 electrodes were excluded for the ELECT-CTL condition. **(c)** Flash response characterization according to Carcieri et al., 2003. *(top)* Spike rasters of a full-field flash stimulus (20 trials, 2 s ON 2 s OFF). *(middle)* Peristimulus time histogram (PSTH) derived from the rasters. *(bottom)* Visual stimulus time course. A_1_ and A_2_ are relative (to baseline) response amplitude for on and off. Tp_1_ and Tp_2_ are time to peak (latency) for on and off. D_1_ and D_2_ are durations for on and off. **(d)** Visual response changes were evaluated by comparing First (red) to Second (blue) and First to Last (green) using the average PSTH binned at 2 ms intervals for all responses. Gaussian smoothing filter *σ* = 4 ms.

In our previous study a detailed description of the electrical stimulation procedure is provided (Material and Methods, Stimulation; Jalligampala et al., 2017). Briefly, the stimulus pulses were generated using a stimulus generator (STG 2008, Multi Channel Systems, Reutlingen, Germany) and were delivered epiretinally via an interior electrode selected near robust neural signals. The stimulus (Figure 1a, left panel) consisted of monophasic rectangular voltage pulses with amplitudes (0.3, 0.5, 1.0, 1.5, 2.0, 2.5 V), included both cathodic (-V) and anodic (+V) stimuli, and durations (60, 100, 200, 300, 500, 1,000, 2,000, 3,000, 5,000 μs). While 0.1 V was also presented, the stimulator was later found to be unable to deliver the waveform faithfully. Accordingly, this stimulus was excluded from analysis. Durations were randomized in 5 sequential sets per voltage, with 5 s intervals between pulses for RGC recovery. Polarity was randomly chosen at the start of each block. Spontaneous activity was recorded for ~30 s before and after each block.

### Visual stimulation

Visual stimuli were projected from below through the transparent MEA using a DLP projector (K10; Acer Inc., San Jose, California, USA). Full-field flashes (~3 × 4 mm) cycled 2 s ON (40 klx) and 2 s OFF (20 lx) for 20 repetitions per block (mean illuminance 20 klx, 99.9% Michelson contrast; Figure 1a). The six visual stimulus blocks were interleaved before, after, and within an electrical stimulation experiment that spanned ~80 min of recording time, including the First and Last flash blocks.

### Test and control conditions

#### Test condition (ELECT-RESP)

RGCs were considered electrically responsive if they met two criteria: (1) ≥ 3 of 96 responses exceeded 2 SD above spontaneous firing rate; (2) firing rate ≥8.89 Hz (≥4 spikes in five 90-ms windows). Only cells recorded on the 8 electrodes surrounding the stimulating electrode (in red, Figure 1b, left panel) were included.

#### Internal control (ELECT-CTL)

This control included electrically nonresponsive cells >300 μm from the stimulating electrode, recorded in the same tissue (Figure 1b, middle panel). A caveat to this control condition is that electrical responses have been shown to extend at least as far as 800 μm from the stimulating electrode (Eickenscheidt et al., 2012; Ryu et al., 2009; Stett et al., 2007; Jalligampala et al., 2017; Wilke et al., 2011).

#### External control (VIS-ONLY-CTL)

This control incorporated cells exposed only to visual stimulation at the same timing as the original protocol. Stimulating visually at the same time points provided a control for any changes occurring during the *in vitro* recording due to all factors excluding electrical stimulation (Figure 1b, right panel).

### Data processing and inclusion criteria

**Figure 2:**
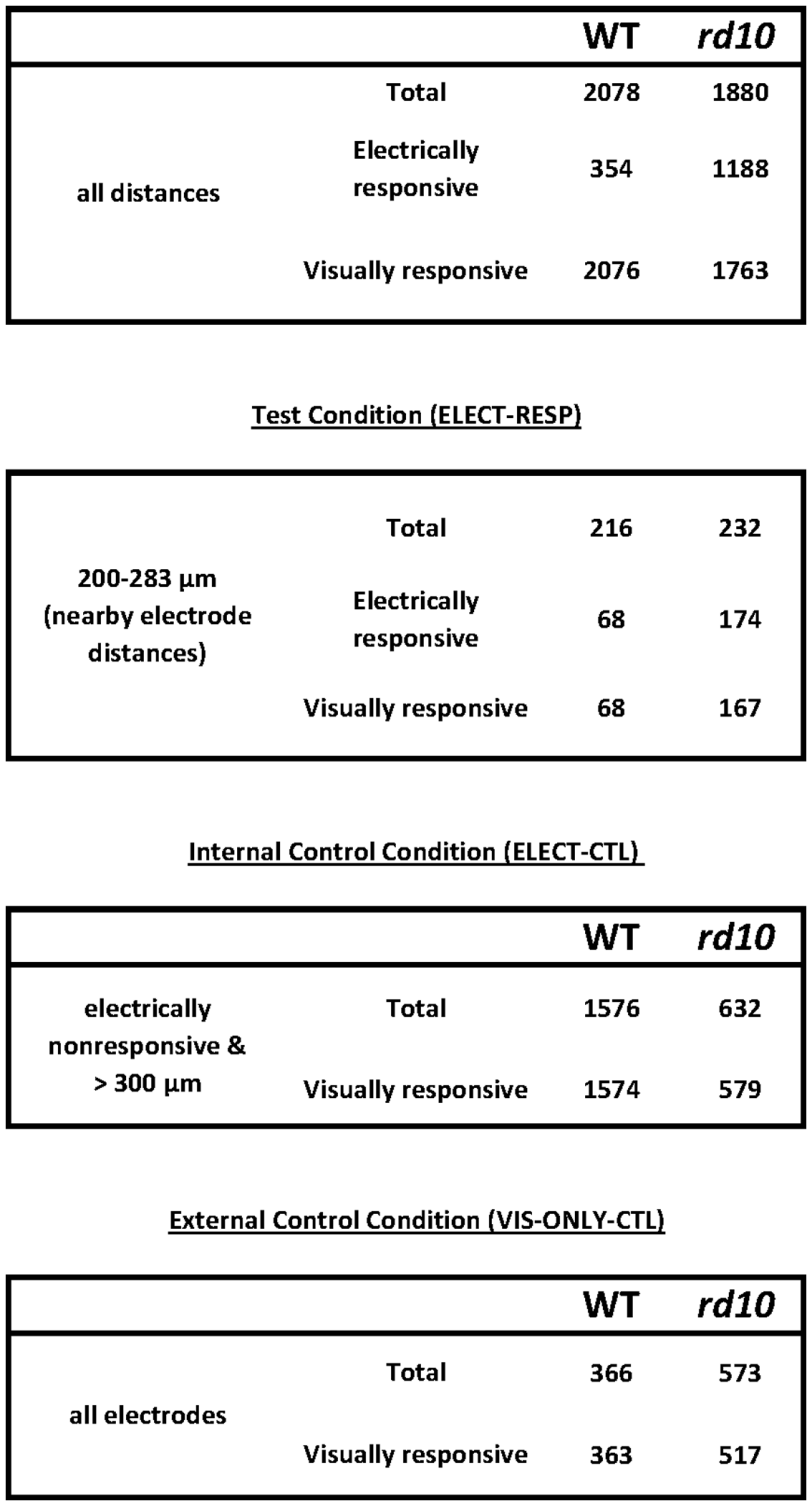
RGC counts for WT and *rd10* for test and control conditions. “distances” are recording distances relative to the stimulating electrode. (For the definition of electrically “responsive” see *Methods: Test and Control Conditions,* and Jalligampala et al., 2017, *Methods–Data Analysis*).

Spike sorting software was used to process raw data (Offline Sorter, Plexon Inc., TX, USA). Raw traces were first filtered using a low-cut, 12-point Bessel filter at 51 Hz to exclude line noise, and thresholded at 4 standard deviations below the mean of the amplitude histogram. Traces were sorted into noise and unit-specific clusters with an automated routine (Standard Expectation Maximization), manually inspected for accuracy, and units were included only if they exhibited: (1) clear lock-out in ISI histogram/autocorrelogram, (2) no peaks in cross-correlograms indicative of split units, (3) distinct principal component separation of biphasic waveforms, and (4) stable waveform shape and firing rate throughout the experiment. The sorted spike timestamps were collected with NeuroExplorer (Plexon Inc., TX, USA) and exported to MATLAB for further analysis. Total RGC counts were 2078 WT cells (16 retinal halves) and 1880 *rd10* cells (9 halves) for test/internal controls; 366 WT cells (3 halves) and 573 *rd10* cells (3 halves) for external controls (Figure 2).

### Data analysis

Determining the visual response parameters: For each visual block (20 repetitions), responses were quantified following Carcieri et al. (2003) (Figure 1c). A peristimulus time histogram (PSTH) was constructed by aligning and averaging the 20 responses (4 s each, 10 ms bins), then smoothing with a Gaussian filter (*σ* = 50 ms). Baseline firing rate and SD were calculated from the last 250 ms of each ON and OFF phase. For each phase, time to peak (Tp_1_, Tp_2_), and baseline-subtracted amplitude (A_1_, A_2_) were measured. Response duration (D_1_, D_2_) was defined as the time for the response to decay from the peak (A) to A/*e* (*e* = Euler’s number). Responses were excluded if amplitude did not exceed baseline + 2 SD or if peaks were likely caused by carryover between phases (latency < 100 ms). Response polarity was quantified using the ON/OFF index: (A_1_ − A_2_) / (A_1_ + A_2_), classifying cells as off (−1 to −0.5), on–off (−0.5 to 0.5), or on (0.5 to 1).

#### Visual response changes

To test whether electrical stimulation alters visual responses, we compared visual response parameters before and after stimulation for the test and both control conditions (Figure 1d). The visual block recorded before any electrical stimulation was defined as the “First” block, and the block recorded after completion of the full protocol (~80 min) as the “Last” block. Because many cells showed changes after only low-voltage stimulation (0.3 and 0.1 V; ~20 min; Figure 1d), the block recorded after this initial period was defined as the “Second” block and used to assess early effects. Although 0.1 V stimuli were delivered, these data were excluded from analysis due to unreliable waveform and charge delivery (Jalligampala et al., 2017).

#### Statistics

To test the hypothesis that visual response parameter medians were unchanged between First and Last as well as between First and Second blocks, Wilcoxon’s *ranksum* test was used (MATLAB; The Mathworks, Natick, MA) at a significance of 0.05. To examine whether significant response parameter changes differed between test and each control condition, Wilcoxon’s *ranksum* test, significance of 0.05, was used to test the hypothesis that the two change distributions have equal medians.

## Results

Here, our goal is to gain a deeper understanding of how ongoing electrical stimulation affects the visual response parameters of different RGC types. In a previous study from our group (Jalligampala et al., 2017), we established an experimental and analysis framework, by which one could identify the optimal stimulus that will activate a majority of RGCs indirectly via network stimulation from the epiretinal side. This stimulus was optimal for “blind” experiments where the specific response properties for each cell were unknown. During the entire duration of the experiment, six visual stimulus blocks of full-field “flash” stimulus were applied to monitor the stability of RGC responses. These visual blocks were interleaved before, after, and between electrical stimulation blocks spanning ~80 min of the entire recording time (Figure 1a). Apart from monitoring the stability of the RGC responses, the visual stimulus provided us with an opportunity to classify the cells into different physiological cell types based on their response to the visual stimulus. Surprisingly, we found evidence that visual responses might change over the course of the experiment. In that previous study, we pooled all the RGCs and did not distinguish them into different physiological cell types. However, in this study, we classified RGCs into different response types to better understand how various visual response parameters change during ongoing electrical stimulation. For this study, the primary data set came from the previous study (Jalligampala et al., 2017) and was used to test our hypothesis that ongoing electrical stimulation alters visual responses in healthy and degenerating mouse retinas. A preliminary version of this study was reported using only that original data (Jalligampala et al., 2015). The additional VIS-ONLY-CTL dataset was collected to account for any non-electrical effects intrinsic to the experimental design.

In an effort to subdivide the RGC population into visual response categories, we examined visual response parameters in the context of a previous study (Carcieri et al., 2003). However, we failed to find strong agreement between our data and the statistically determined response distribution boundaries that delineated types in that study (see Supplementary Figures S1, S2). Nevertheless, due to the predominance of the on, off, and on- off types in the established literature (Morgan and Wong, 2007), we divided our data according to the approximate ON/OFF index boundaries of Carcieri et al. (2003) (see *Methods*).

### Diversity in alteration of visual responses to electrical stimulation

**Figure 3:**
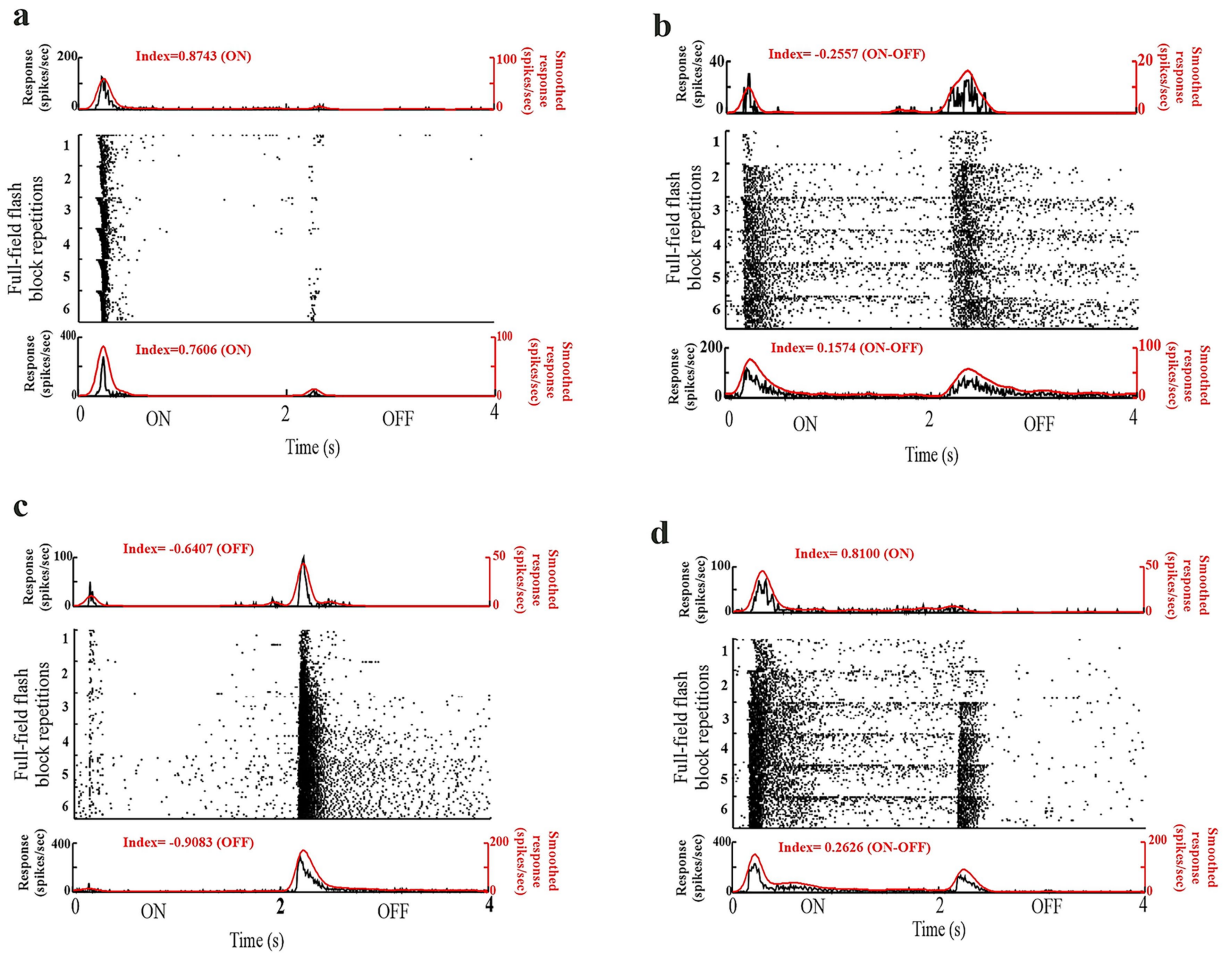
Diversity of visual response changes. **(a–d)** Rastergram depicts responses for all six visual blocks. Response differences between blocks 1 (First), 2 (Second), and 6 (Last) were examined. *(Top)* Average PSTH for First block. *(Bottom)* Average PSTH for Last block.

To evaluate how electrical stimulation alters the visual response parameters, we plotted the rastergram and peristimulus time histograms (PSTHs) of the cell’s response to full-field flash stimulus (Figure 3). These example cells were both electrically and visually responsive. The rastergram shows the visual response to all six flash blocks presented before, after, and between the electrical stimulation blocks. The PSTH (above) represents the average response (20 trials) of the “First block,” i.e., before electrical stimulation. The PSTH (below) shows the average response (20 trials) of the “Last block,” i.e. after the entire electrical stimulation protocol was over. We observed diversity in the alteration of the visual responses. For our study, our definition of neural adaptation was a change in the cell’s firing rate. Therefore, to evaluate the effect of electrical stimulation, we observed how the cell’s firing rate changed following a period of electrical stimulation. Apart from the changes in firing rate, shown in all the example cells (Figures 3a–d), we also observed changes in other visual response parameters (latency, duration, and ON/OFF index). For some cells, we observed that the latency of the responses became shorter following electrical stimulation (Figure 3d, on response). For some cells, we observed a change in the transiency of the responses, e.g., some cells which were transient before electrical stimulation became sustained following electrical stimulation (Figures 3c,d). Interestingly, we observed for some cells, a change in ON/OFF index, i.e., before electrical stimulation the cell responded to a single phase of the flash stimulus, however after electrical stimulation the cell responded to both phases of the flash stimulus (Figure 3d, on to on–off). Similar heterogeneous changes in response properties were also observed in the VIS-ONLY-CTL condition, indicating that visual stimulation alone can produce varied adaptation effects even in the absence of electrical stimulation.

**Figure 4:**
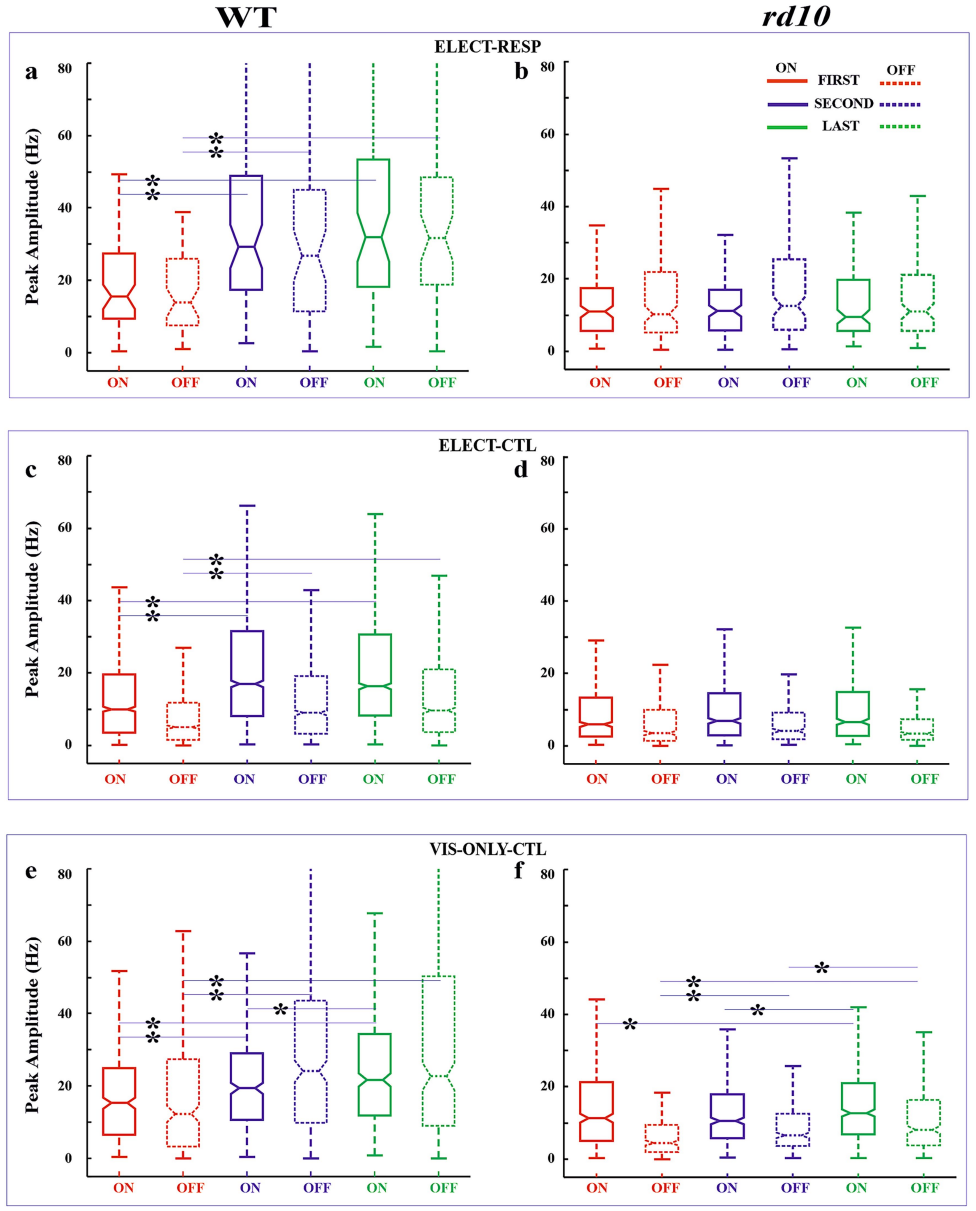
Box-whisker plots for response amplitude. Box plots for on (solid line) and off (dashed) responses shown for first (red), second (blue), and last (green) flash blocks for WT **(a,c,e)** and *rd10* **(b,d,f)** retinas for the test (ELECT-RESP, **(a,b**) and control [ELECT-CTL, **(c,d)** and VIS-ONLY-CTL, **(e,f)** conditions]. Horizontal lines of the box plot demarcate 25, 50, and 75% quartiles. Notches are 95% confidence intervals for the median (50% quartile). Whiskers denote data range excluding outliers. Some outlier cutoffs are clipped to show detail. Refer to Figure 7 for pairwise statistical tests. Asterisks: significantly different distributions for FIRST-SECOND, FIRST-LAST, and SECOND-LAST comparisons—*p*-values in Figure 7.

**Figure 5:**
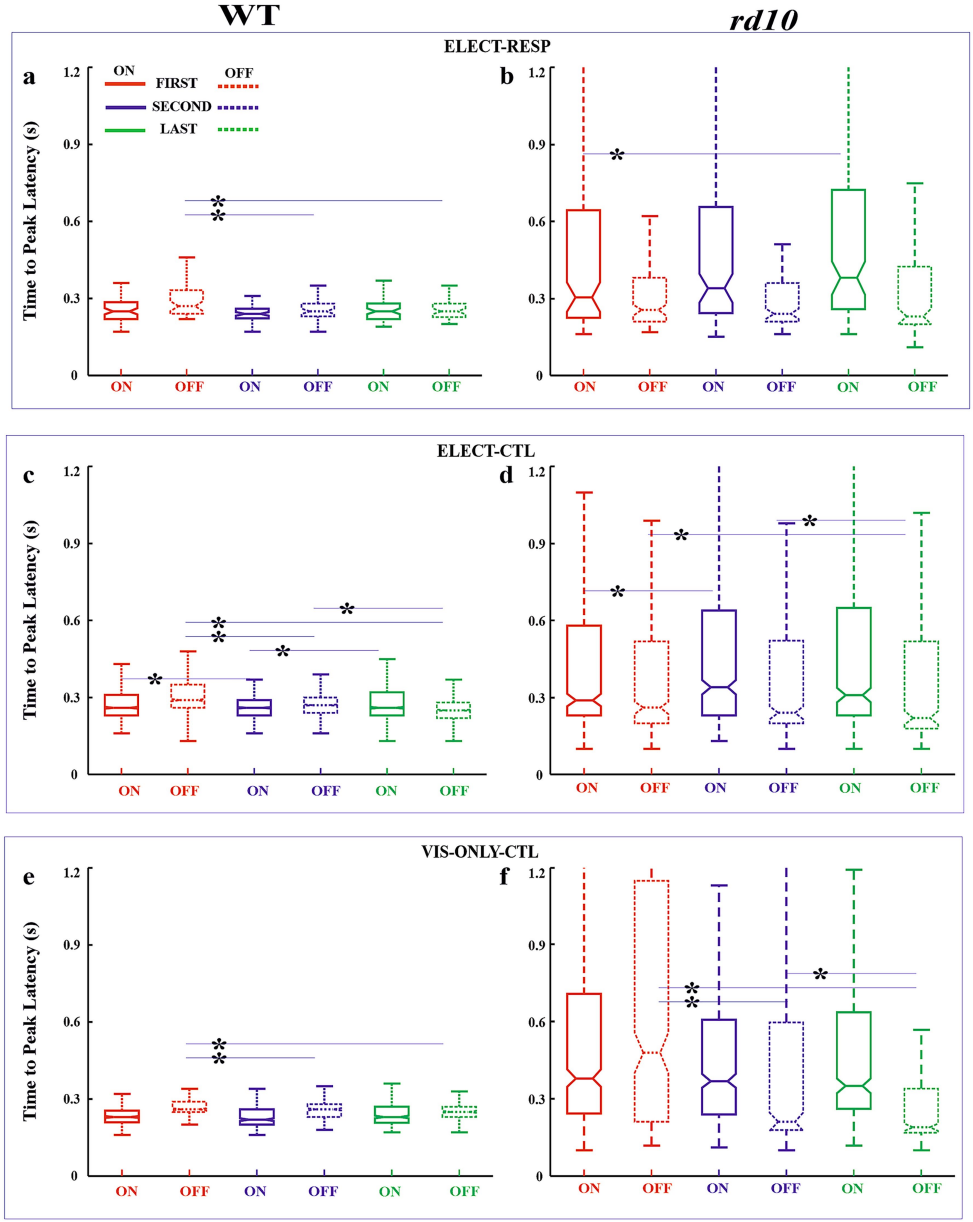
Box-whisker plots for response latency. Box plots for on (solid line) and off (dashed) responses shown for first (red), second (blue), and last (green) flash blocks for WT **(a,c,e)** and *rd10* **(b,d,f)** retinas for the test [ELECT-RESP, **(a,b**) and control [ELECT-CTL, **(c,d)** and VIS-ONLY-CTL, **(e,f)** conditions]. Horizontal lines of the box plot demarcate 25, 50, and 75% quartiles. Notches are 95% confidence intervals for the median (50% quartile). Whiskers denote data range excluding outliers. Some outlier cutoffs are clipped to show detail. Refer to Figure 7 for pairwise statistical tests. Asterisks: significantly different distributions for FIRST-SECOND, FIRST-LAST, and SECOND-LAST comparisons—*p*-values in Figure 7.

**Figure 6:**
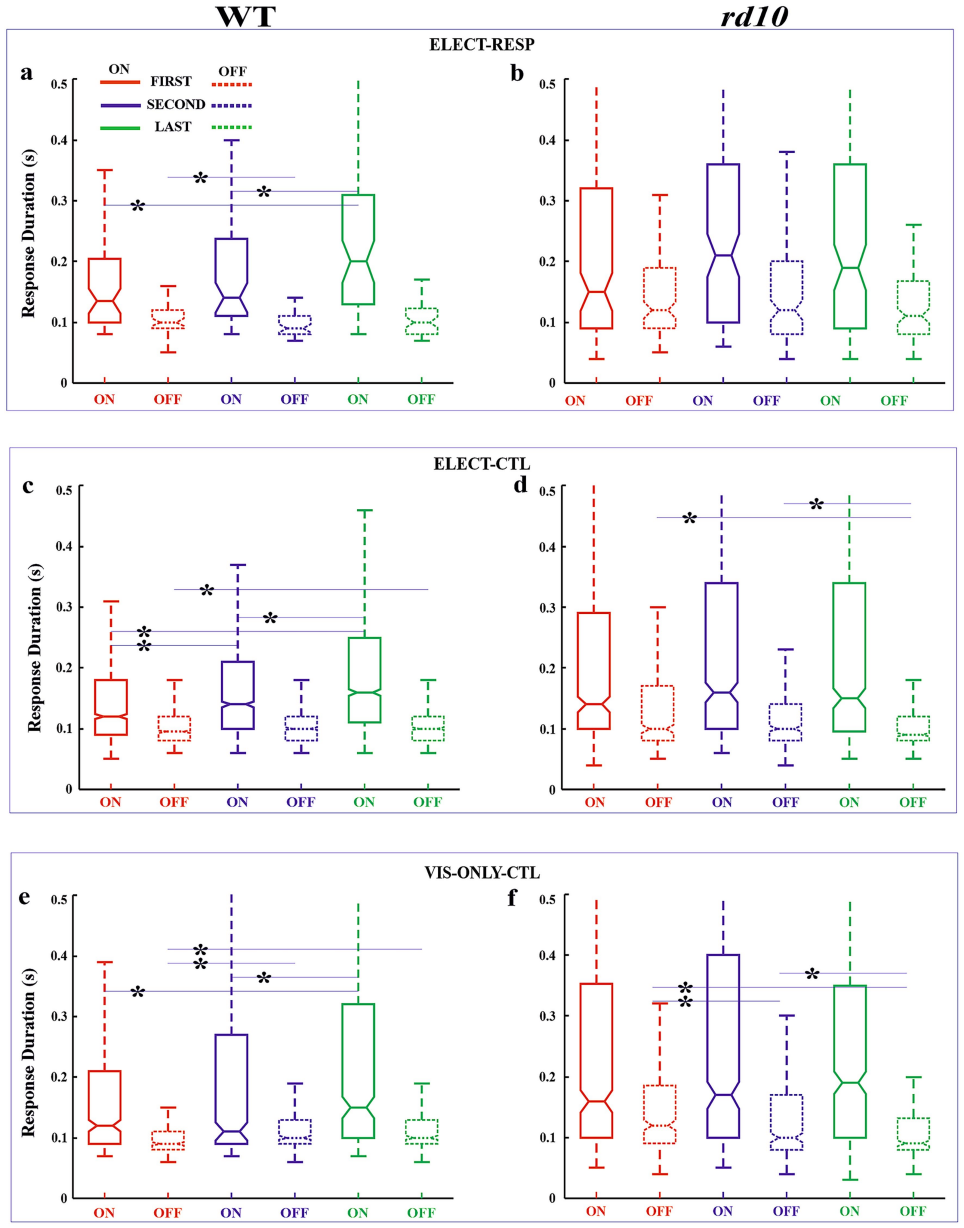
Box-whisker plots for response duration. Box plots for on (solid line) and off (dashed) responses shown for first (red), second (blue), and last (green) flash blocks for WT **(a,c,e)** and *rd10* **(b,d,f)** retinas for the test [ELECT-RESP, **(a,b**) and control [ELECT-CTL, **(c,d)** and VIS-ONLY-CTL, **(e,f)** conditions]. Horizontal lines of the box plot demarcate 25, 50, and 75% quartiles. Notches are 95% confidence intervals for the median (50% quartile). Whiskers denote data range excluding outliers. Some outlier cutoffs are clipped to show detail. Refer to Figure 7 for pairwise statistical tests. Asterisks: significantly different distributions for FIRST-SECOND, FIRST-LAST, and SECOND-LAST comparisons—*p*-values in Figure 7.

**Figure 7:**
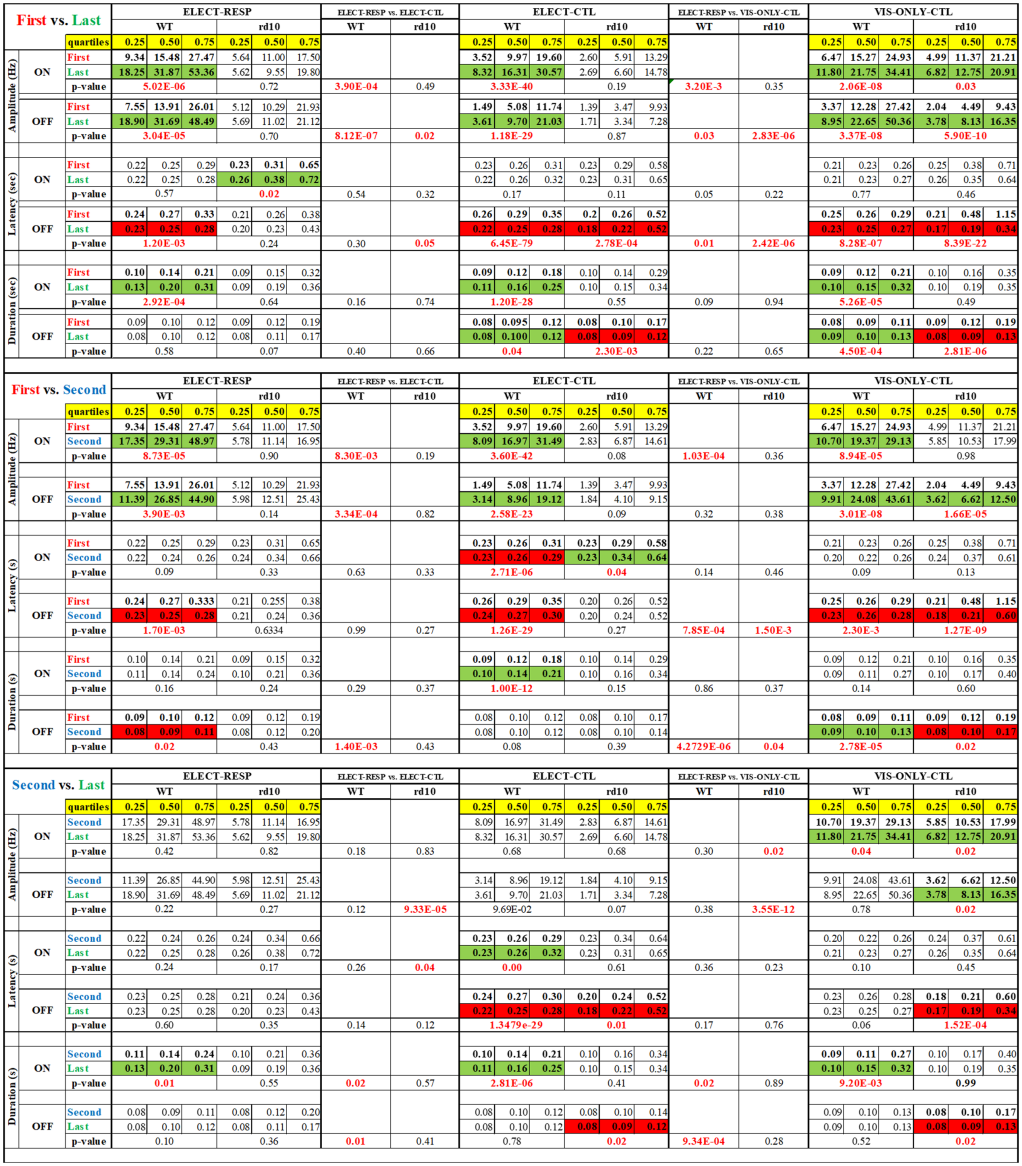
For the plots in Figures 4–6, the nonparametric Wilcoxon’s *ranksum* test (MATLAB, *p* < 0.05) was used to determine significant changes. For each condition (responsive and nearby ELECT-RESP, nonresponsive and distant ELECT-CTL, and the visual-only control VIS-ONLY-CTL) and each mouse strain (WT and *rd10*) we tested whether each visual response parameter differed between First vs. Second, First vs. Last, and Second vs. Last flash stimulus blocks. For each of these tests, the quartiles of the First, Second, and Last response parameter distributions are provided along with the p-value of the test. For testing whether the parameter changes were significantly different between test and control conditions, p-values are presented before the middle and right data blocks. Green boxes identify significant increase. Red boxes identify significant decreases. Bold p-values highlight significant changes. Red p-values indicate significance between test and control conditions.

As stated, our primary measure of visual response adaptation to electrical stimulation was the change in firing rate, quantified by on and off response amplitudes. We also analyzed changes in response latency and duration. These parameters are shown in notched box-whisker plots (median, 95% CI, quartiles, and outliers) to illustrate variability across conditions (Figures 4–6). We first compared responses between the first and last blocks. Because changes appeared as early as ~20 min (second block, after 0.1 and 0.3 V stimulation), we also compared the first and second blocks. Finally, to assess slower adaptation, we compared the second and last blocks (~60 min apart). All comparisons were made between the test condition (ELECT-RESP) and control conditions (ELECT-CTL and VIS-ONLY-CTL; see Methods). Statistical results are shown in Figure 7, cell numbers in Supplementary Figure S6, and outliers in Supplementary Figures S3–S5. Supplementary Figure S8 has been added to show the difference in response amplitude medians between ELECT-RESP and each of the control conditions. The main results are as follows. In all three conditions, WT cells showed increased firing rates to both light onset and offset, with increased response duration to light onset. Amplitude increased shortly after electrical stimulation, whereas duration increased significantly only later, and both effects were stronger in electrically responsive cells. In contrast, such adaptation was not consistently observed in *rd10* retina. Other changes in latency and duration occurred but did not show a strong or consistent effect of stimulation. Notably, in *rd10* retina under control conditions, the latency and duration of light-offset responses decreased over time, though not consistently relative to the stimulation condition. A detailed analysis follows.

#### WT retinas

1. *First vs. Last Block–*Amplitude: For both ON and OFF responses, relative amplitude increased significantly from the First to the Last block across all three conditions (Figures 4a,c,e). However, this increase was significantly greater in the test condition (ELECT-RESP) than in the controls (ELECT-CTL, VIS-ONLY-CTL; Figure 7), indicating that electrical stimulation enhances the amplitude increase beyond visual stimulation alone. Latency: ON response latency showed no significant change across conditions. OFF response latency decreased significantly in all three conditions, with a slightly greater decrease in ELECT-RESP compared to VIS-ONLY-CTL, but no difference between ELECT-RESP and ELECT-CTL (Figures 5a,c,e). Duration: ON response duration increased significantly in all three conditions, with no significant differences between test and controls (Figures 6a,c,e). OFF response duration increased significantly only in the control conditions, with no significant effect of electrical stimulation.
2. *First vs. Second Block–*Amplitude: For both ON and OFF responses, there was a significant increase in the relative amplitude from the First to the Second block for all three conditions. When comparing the magnitude of pre- and post-stimulation changes between the test and control conditions, we found the increase in ON amplitudes to be significantly greater for ELECT-RESP. However, the increase in OFF response amplitudes was only significantly greater for ELECT-RESP in comparison to the ELECT-CTL, but not VIS-ONLY-CTL (Figures 4a,c,e). Latency: The latency of the ON responses significantly decreased from the First to the Second block only for ELECT-CTL, and this change was not significantly different when compared to ELECT-RESP (Figures 5a,c,e). For the OFF response latency, there was a significant decrease in latency from the First to the Second block in all conditions. On comparing the test and control conditions, this decrease was significantly greater for ELECT-RESP compared to VIS-ONLY-CTL, but not ELECT-CTL. Duration: There was a significant increase in duration of the ON responses from the First to the Second block only for ELECT-CTL. For the ELECT-RESP, there was a significant decrease in duration of the OFF responses from the First to the Second block, and this change was significant compared to the unchanged OFF duration of ELECT-CTL and the increased OFF duration of VIS-ONLY-CTL (Figures 6a,c,e).
3. *Second vs. Last Block -* Amplitude: The only amplitude change from the Second to the Last block was for the on response amplitude of VIS-ONLY-CTL. However, comparing this to ELECT-RESP, there was no statistical significance (Figures 4a,c,e). Latency: Only the ELECT-CTL condition exhibited latency changes from Second to Last block. While the on response latency increased, the off latency decreased (Figures 5a,c,e). Duration: There was a significant increase in duration of the on responses for all three conditions. Furthermore, comparing the test to the control conditions the magnitude of increase in on response duration was significantly greater for the ELECT-RESP (Figures 6a,c,e). For all three conditions, there was no significant change in off duration from the Second to Last block.

#### *rd10* retinas

1. *First vs. Last Block–*Amplitude: The only significant amplitude change from First to Last block was an increase in on and off response amplitudes for VIS-ONLY-CTL (Figures 4b,d,f). This increase was only significantly different from ELECT-RESP for the off response. Latency: There was a significant increase in on response latency from the First to Last block for ELECT-RESP. However, this was not a significant change compared to the control conditions. For both control conditions, there was a significant decrease in off response latency from the First to the Last block, and this change was also significant relative to the test condition (Figures 5b,d,f). Duration: The only response duration changes from the First to the Last block were significant decreases in the duration of off responses for both control conditions. However, compared to the test condition these changes were not significant (Figures 6b,d,f).
2. *First vs. Second Block–*Amplitude: The only change in amplitude from the First to the Second block was for the off response of VIS-ONLY-CTL, but this change did not differ from the ELECT-RESP (Figures 4b,d,f). Latency and Duration: For the on response of ELECT-CTL latency increased from the First to Second block, but was not significant relative to ELECT-RESP. In contrast, both off response latency and duration decreased for VIS-ONLY-CTL. While both decreases were significant relative to the ELECT-RESP, only the decrease in off latency was large in magnitude (Figures 5, 6b,d,f).
3. *Second vs. Last Block–*Amplitude: The only significant amplitude changes from the Second to the Last block were increases in on and off response amplitudes for VIS-ONLY-CTL. While modest, the magnitudes of these changes were significant relative to the ELECT-RESP (Figures 4b,d,f). Latency and Duration: Both latency and duration of the off responses for both control conditions decreased from the Second to Last block. Nevertheless, the magnitudes of these changes were small and not significant in comparison to the test condition (Figures 5,6b,d,f).

### Change in the ON/OFF index

**Figure 8:**
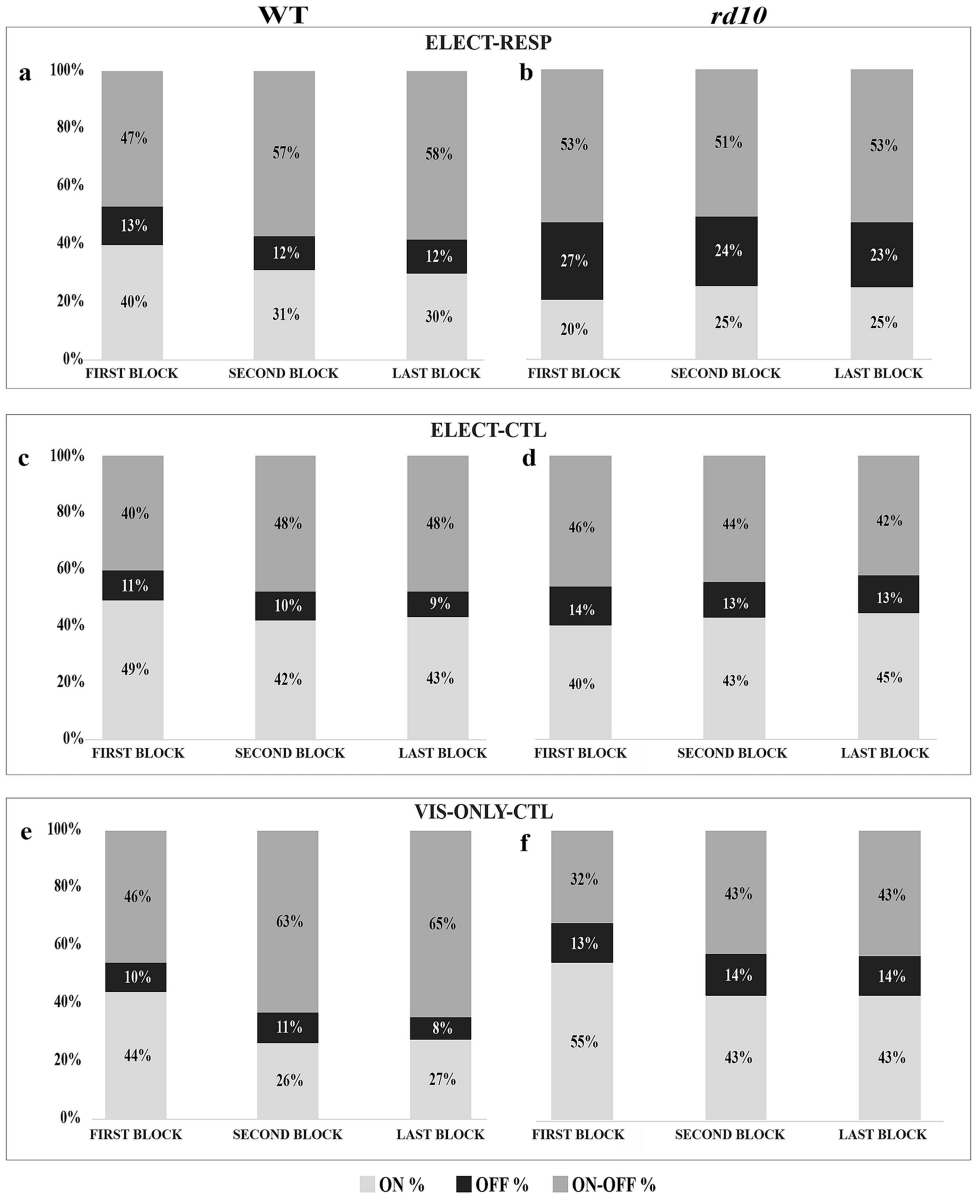
Changes in ON/OFF index. Percentage bar shows the relative proportion of on, off, and on–off responses for first, second, and last block, for WT **(a–c)** and *rd10* retina **(d–f)** for all the three conditions (ELECT-RESP, ELECT-CTL, and VIS-ONLY-CTL). Corresponding cell counts are provided in the Supplementary Figure S7.

In Figure 8 we examined the proportions of RGCs classified as on, off, and on–off before and after electrical stimulation for the test and control conditions. For the WT retinas, we observed a change in the distribution of ON/OFF index values from the First to the Second block for all three conditions. This change in ratio was primarily the change from the purely on (0.5 to 1) to on–off (−0.5 to 0.5) responses. Given that this change in ON/OFF index was similar for all three conditions, we surmise that this change in weighting of the on and off responses is primarily caused by adaptation of the WT retina to visual stimulus. Furthermore, such adaptation is rather fast as seen by the change from the First to the Second block, but not to the Last block for all the conditions (Figures 8a,c,e). Interestingly, for the *rd10* retinas, the above observation (on to on–off) was observed only in the absence of electrical stimulation (VIS-ONLY-CTL, Figure 8f). For both the ELECT-RESP and the ELECT-CTL conditions we saw no change in the ON/OFF index distribution between the three visual blocks (Figures 8b,d). This might suggest that the presence of electrical stimulation prevents a change in the ON/OFF index in the *rd10* retinas regardless of whether or not the RGCs are electrically responsive. Also of interest for the *rd10* retina, we found that the relative proportion of off cells was greater for the population of RGCs that were responsive to electrical stimulation (ELECT-RESP), but not for the nonresponsive population (ELECT-CTL). We interpret this to reflect that, in the *rd10* retina, the relative sensitivity of off and on cells to electrical stimulation is altered to favor off cells. The cell numbers contributing to each visual block for the test and control conditions are presented in the Supplementary Figure S7.

## Discussion

In the present study, we focused on discerning the shifts in mouse retinal ganglion cell (RGC) visual response parameters —specifically, latency, duration, and amplitude— before and after electrical stimulation. Our findings illuminate a noteworthy trend: adaptation to visual stimulation *in vitro* involves increased neuronal responsiveness as evidenced by increased firing rate (amplitude) of the cell. Electrical stimulation further strengthens these visual response changes. We are cautious, however, not to draw any further strong conclusions from the changes observed. Given the extensive scope of our analysis, involving nearly 200 statistical comparisons, and the potential for approximately a dozen instances of spurious significance among the 75 observed adaptational and between-condition changes, we recognize the inherent complexity of interpreting such data. For this reason, we have meticulously reported actual *p*-values and restricted our attention to the most robust adaptation patterns, ensuring a prudent approach to our conclusions.

### Response changes of retinal ganglion cells in *rd10*

In retinitis pigmentosa, the loss of photoreceptor cells disrupts the normal functioning of on and off pathways, leading to alterations in adaptation mechanisms. Individuals with RP experience difficulties in adapting to changes in lighting conditions, as well as decreased contrast sensitivity and impaired night vision (Daiger et al., 2013; Sahel et al., 2015, 2021). This highlights the importance of understanding adaptation mechanisms in retinal degeneration. Research on the adaptation of degenerated mouse retina has revealed several intriguing findings, including functional remodeling, enhanced sensitivity, and plasticity in inner retinal circuits. After the loss of photoreceptor cells, other retinal cell types undergo functional remodeling to compensate for the decreased input from photoreceptors (Jones et al., 2016). This remodeling process involves changes in synaptic connectivity, neurotransmitter release, and receptive field properties of retinal neurons. Degenerated retinas often exhibit enhanced sensitivity to dim light stimuli, possibly due to changes in the gain control mechanisms within the retinal circuitry (Stasheff, 2008). After rod degeneration in rd10 mice, bipolar and horizontal cells retract dendrites and sprout ectopic processes, while Müller glia become reactive, hallmarks of retinal remodeling in retinitis pigmentosa (Phillips et al., 2010). These changes alter synaptic connectivity and the balance of excitatory/inhibitory inputs to RGCs, modifying network gain. Lin and Peng (2013) have demonstrated plasticity in inner retinal circuits of degenerated retinas, with surviving retinal neurons exhibiting increased dendritic arborization and synaptic remodeling. This structural plasticity may contribute to the functional adaptation of degenerated retinas to changes in visual input.

In rd10 retinas, normal energy production pathways are disrupted, with altered purine/pyrimidine and mitochondrial metabolites indicating metabolic reprogramming during degeneration (Pan et al., 2021). Oxidative stress suppresses key glycolytic enzymes such as pyruvate dehydrogenase and pyruvate kinase, reducing glucose utilization and shifting flux toward the pentose phosphate pathway, reflecting a stressed metabolic state and altered redox balance (Kanan et al., 2022). These changes are accompanied by increased oxidative damage and imbalances in antioxidant defenses, occurring alongside structural remodeling (Kushwah et al., 2023). Such metabolic rewiring may influence how remaining circuits adapt or fail to adapt to ongoing stimulation and could contribute to the counteracting effect of electrical stimulation on visual adaptation in rd10 retinas.

For our *rd10* dataset, we observed the majority of adaptational changes for off responses. Furthermore, almost all changes were only found in control conditions, suggesting that electrical stimulation might antagonize visual adaptation in the *rd10* retina. A previous study has shown that with progressive degeneration in *rd10* retinas both the on and off responses are equally affected (Stasheff et al., 2011) However, for retinas of the rd1 mouse strain, which is an aggressive form of retinal degeneration, the off pathway remains preserved for a longer time span in comparison to the on pathway (Stasheff, 2008). In contrast, even more recent research indicates that the OFF responses are more sensitive to degeneration (Cha et al., 2022; Dyszkant et al., 2024). Regardless, our observations seem to support the notion that retinal degeneration has differential effects on the on and off pathways and their adaptations (Stasheff, 2008).

### Role of *in vitro* adaptation

It is often observed that over the course of long-term *in vitro* recordings the physiological properties of the tissue change (sometimes called “experimental run-down”). Such changes can lead to changes in visual as well as electrical responses. For example, a recent study showed that increased temperature caused the electrically elicited RGC responses to be more stimulus-locked, with shorter latency and shortened durations (Maturana et al., 2015). While such changes could result from changes in pH, temperature, and/or oxygenation, we controlled for these variables in our experiments. Nevertheless, other variables such as metabolic waste buildup or depletion of cellular resources like glutamate precursors still lead to gradual response changes that eventually conclude with the metabolic death of the tissue. Therefore, to account for such changes during *in vitro* recording, we compared changes associated with electrical stimulation to an external control condition (VIS-ONLY-CTL) in which the same run-down effects and minimal light stimulation (~4 of 80 min) were present.

### Contribution from visual adaptation

Work in human ERG (electroretinogram) has shown a gradual increase in amplitude of the a-wave and b-wave components during adaptation to strong light stimulation over a period of approximately 20 min, suggesting that the photoreceptor changes are involved in the rise in amplitudes (Gouras and MacKay, 1989). This observation could explain the immediate response changes (~20 min) observed even in the VIS-ONLY-CTL condition. This increase in response is thought to reflect the redepolarization of the cones, after the initial hyperpolarization to an adaptation field. Such redepolarization also restores the horizontal cell polarization. Therefore, apart from any changes induced by the *in vitro* environment, adaptation to visual stimulus also plays a role in the increased response in all three conditions (Webster, 2015).

### Characterizing visual cell type before electrical stimulation

We have shown in this study that electrical stimulation can alter visual responses. Therefore, when visual stimulation is to be used for characterizing RGC type, it is strongly recommended that such characterization is performed before any electrical stimulation has been presented. Such characterization is most relevant in healthy retinas or early-stage degenerating retinas, as it is tough to classify the cells during the late stages of degeneration when the visual responses are mostly lost. In addition to visual cell typing, patch-clamp or calcium imaging methods can identify individual cell types morphologically and electrophysiologically. However, it remains untested how electrical stimulation might influence such methods. Recent work from our group employs a newer method to differentiate dozens of visual response types (Shabani et al., 2024). Notably, this work also shows signs of response changes following electrical stimulation (see below).

### ON/OFF index changes in healthy and *rd10* RGCs

In our study, we observed a change in relative weighting for on and off responses, with a shift of purely on responses to on–off. This shift was mostly observed in WT retinas for all three conditions and in the *rd10* retinas only for the VIS-ONLY-CTL condition. Similar observations have been reported in a previous study in healthy retinas (Tikidji-Hamburyan et al., 2015). This prior study showed that, upon full-field stimulation at certain light levels, a cell might be classified as off, while at other light levels it would be classified as on–off. Together these observations demonstrate that the relative weighting of on and off response contributions is not fixed and that it can be altered by adaptational mechanisms. In our study, these changes primarily occurred from the First to the Second block (20 min) and remained constant through the Last block (80 min). One possible reason for this observation is that the first visual stimulus is presented after 30 min of no light stimulation (dark adapted state), however, after electrical stimulation and *in vitro* adaptation to periodic light stimulation the retina was in a different adaptational state. For *rd10* retina, however, we did not observe such changes in the test (ELECT-RESP) and internal control (ELECT-CTL). A possible reason may be that the electrical stimulation is antagonistic to visual adaptation in the degenerating retina. Subsequent experiments should examine how visual and electrical adaptation interact in the healthy and degenerating retina at multiple time points during the course of degeneration, although some researchers have already begun to probe this topic (Arens-Arad et al., 2020; Arens-Arad et al., 2021; Ly et al., 2025).

**Figure 9:**
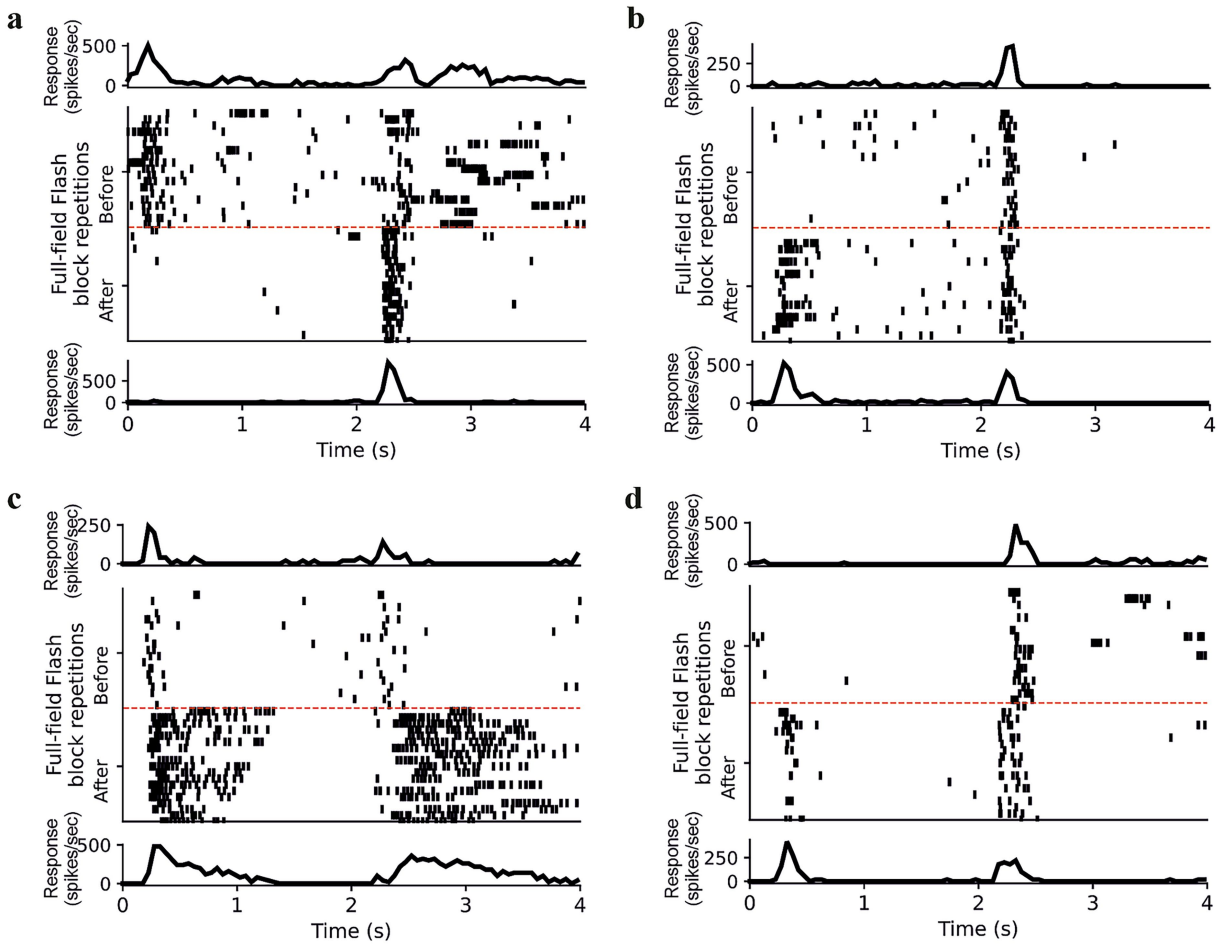
Visual response changes from a separate study. **(a–d)** Rastergram depicts responses for a visual stimulus block before and one after at least 30 min of electrical “white noise” stimulation. *(Top)* Average PSTH for block before electrical stimulation. *(Bottom)* Average PSTH for block after. Experimental protocol is elaborated in *Methods*—*Electrical Stimulation* and (Shabani et al., 2024).

In more recent work from our lab, we have continued to observe visual response changes in the context of a different, ongoing electrical stimulation (Shabani et al., 2024). Rather than isolating electrical pulses by 5 s, this newer study presented them at a rate of 25 Hz continuously for minutes at a time. The pulse amplitudes spanned a similar range; but were concentrated as a mostly subthreshold Gaussian distribution with mean of −800 mV and SD of 280 mV. Because pulse amplitudes were pulled from this distribution randomly, the stimulus was termed “electrical white noise”—in analogy to similar visual and auditory white noise stimuli that are commonly used in sensory neuroscience research. In Shabani et al., 2024, visual response changes were also observed in ON/OFF index, duration, latency, and peak amplitude (Figure 9). The persistence of such visual response adaptation, even for ongoing electrical stimulation that was designed to better match bionic vision implementations, reinforces a need to better understand the relevance of RGC adaptation to bionic vision.

### Potential cellular mechanisms underlying adaptation

In this study, we have noted changes in visual responses (1) during visual stimulation alone, (2) with additional electrical stimulation, and (3) in the additional context of the degenerated retina. We have cautiously used the term adaptation since we have examined and described these response changes in the context of experimentally controlled stimuli. In contrast, the slower acting mechanisms of homeostatic plasticity are unlikely to influence our results in the healthy retina (Fitzpatrick and Kerschensteiner, 2023). Most response changes we observed (for both visual-only and electrical stimulation) happened between first and second blocks of visual stimulation - separated by about 20 min. Fewer additional or further response changes were observed between second and last blocks—separated by about 60 min. Conversely, the homeostatic mechanisms affecting intrinsic excitability, synaptic connectivity, and neurite coverage are believed to require hours to months to take effect. Therefore, we suspect most observed changes resulted from more traditional adaptational mechanisms. Nevertheless, differences in adaptation observed between WT and *rd10* retinas may derive in part from the engagement of homeostatic plasticity mechanisms in the degenerating retina. As an example, we saw amplitude increases from first to second block that were significant relative to controls for WT, whereas such increases were significant in *rd10* retina only from first to last block or second to last block. This may point to homeostatic scaling in the degenerating retina attenuating or slowing down adaptational mechanisms.

This framework is doubly attractive in that it leads us to differentiate between adaptation to direct stimulation (light for photoreceptors, and electricity for each of the retinal neuron types) and adaptation to upstream neuronal activity which is a shared mechanism in bipolar cells and RGCs for both light and electrical stimulation.

One adaptational mechanism that could underlie the strengthening of visual responses involves changes in synaptic gain within inner retinal circuits. In many forms of visual adaptation, presynaptic bipolar and amacrine cell synapses adjust their output strength based on recent activity, leading to facilitation or depression of excitatory and inhibitory input onto RGCs (e.g., contrast gain control via bipolar synapse depression and amacrine-mediated modulation) (Nikolaev et al., 2013). Complementary to this, intrinsic changes in RGC excitability can also contribute to adaptive shifts; for example, modulation of ion channel availability. For example, slow inactivation of sodium channels or after-hyperpolarization mechanisms can alter a ganglion cell’s spike generation properties following sustained stimulation, thereby affecting its responsiveness independent of synaptic input (Demb, 2008). These two mechanisms are likely to act in concert to shape the adaptive state of RGCs in response to ongoing visual or electrical stimulation.

### Homeostatic plasticity and ON/OFF pathway balance

Emerging evidence suggests that homeostatic plasticity can be adaptive, helping to preserve retinal function despite degenerative challenges such as inherited retinal degenerations and age-related macular degeneration (Fitzpatrick and Kerschensteiner, 2023). In a mouse model of retinitis pigmentosa (P23H rhodopsin mutation), researchers found large-scale changes in gene expression resembling a developmental state, along with strengthened signaling between rods and rod bipolar cells after rod loss. Remarkably, these mice maintained highly sensitive night vision even after losing more than half of their rod photoreceptors (Leinonen et al., 2020). When ON-pathway input was partially reduced, retinal ganglion cells (RGCs) engaged helpful compensatory mechanisms, such as increasing excitatory synaptic protein expression, which supported the maintenance of visual function. In contrast, complete loss of ON input caused maladaptive changes in the cells’ intrinsic electrical properties and produced unstable activity in some OFF-type RGCs, ultimately degrading visual processing. ON-pathway suppression also changed how the OFF pathway encoded luminance and contrast (Khoussine et al., 2025). In young mouse retinas, most bipolar cell types compensated for the loss of half their cone input by forming new synaptic connections or expanding dendrites, thereby preserving normal visual function. In mature retinas, this homeostatic plasticity was largely absent, resulting in weaker bipolar cell light responses and impaired vision, demonstrating that the capacity for rewiring declines markedly with age (Shen et al., 2020). These processes provide a theoretical framework for interpreting our finding that ongoing electrical stimulation can modulate visual adaptation: the retinal network itself is not static but engages intrinsic and synaptic compensatory mechanisms that shape response strength and balance in both healthy and degenerating retina.

### Limitations

For the current study, we used a simplified full-field stimulus. With this simplification we could gather responses from thousands of RGCs more quickly, however our ability to unambiguously categorize the visual response of individual cells was diminished. For example, if an on cell has a strong enough off surround, it may be categorized as on–off or even off with the present stimulus. Similarly, response latencies and durations can be contaminated when a full-field stimulus is used. Therefore, using stimulation spots to elicit a response primarily from the visual receptive field center would help in classifying the cell’s response more accurately, but would require streamlined methods for identifying receptive field centers in up to 100 RGCs recorded simultaneously. The use of more localized stimuli would also enable examination of whether center and surround mechanisms respond differentially to the adaptation described here.

This study focused on voltage-controlled stimulation and underlying changes in healthy and *rd10* retinas. However, voltage- and current- stimulation may activate different types of retinal neurons in different ways, leading to distinct adaptation profiles. While a monophasic voltage pulse generates a biphasic current pulse, the exact current waveform generated differs somewhat from that generated under current control. Nevertheless, the underlying mechanisms of adaptation are likely conserved across stimulation modalities, as they depend on the cumulative activity of the retinal circuitry rather than the specific waveform. Therefore, we expect that the qualitative trends observed here, such as strengthened responses in WT retinas and counteracting effects in rd10 retinas, would also manifest under biphasic, current-controlled stimulation, though the exact amplitudes, latencies, and recruitment patterns may differ (Fried et al., 2006; Jensen and Rizzo, 2007; Lorach et al., 2015). Additionally, the spatial distribution of electrical stimulation may also influence adaptation mechanisms in the retinal network. These issues also warrant further investigation. Although our study used monophasic, voltage-controlled pulses, biphasic, current-controlled pulses more commonly used in implants may engage retinal neurons differently due to charge balancing and current spread.

### Implications to the field of retinal prostheses

The observed increase in response amplitude is likely the result of alterations in the weighting of network inputs and/or cell-specific physiological properties. Such changes may also affect how the RGCs respond to electrical stimulation of the retinal network. Such effects may have been overlooked in earlier studies since few investigators habituate the retina to ongoing electrical stimulation before examining its electrical responsiveness. An investigation of the cellular and network changes induced by ongoing electrical stimulation—particularly focusing on electrical responsiveness will shed light on this possibility. Given the likelihood that ongoing electrical stimulation will be shown to influence cellular and network responsiveness, and the reality that applied visual prosthetics involve ongoing stimulation, it is advisable to develop paradigms of ongoing background electrical stimulation for future investigations of prosthetic retinal stimulation. One method we have developed is to provide the retina with an ongoing electrical noise stimulus (Sekhar et al., 2016). Such stimulation allows electrical responsiveness to be examined in a steady state of adaptation to electrical stimulation that more closely matches the real-world implementation of retinal prosthetic implants.

## Data Availability

The raw data supporting the conclusions of this article will be made available by the authors, without undue reservation.
