# Supplementary material for: Adaptation of visual responses in degenerating rd10 and healthy mouse retinas during ongoing electrical stimulation
Source: Front Neurosci. 2026 Mar 25;20:1730445. doi: 10.3389/fnins.2026.1730445 (PMC13022506; doi:10.3389/fnins.2026.1730445)
Supplement: Supplementary file 1 [file Data_Sheet_1.DOCX]

# Supplemental Materials

## Overall Distribution of Response Parameters

Using full-field flash stimulus, we evaluated the distribution of visual response parameters. The response parameters measured were the latency of the response to light on (on) and light off (off), duration of the response to on and off and the relative amplitudes of the response to on and off (*see Data Analysis*). Distributions were examined for both the test (ELECT-RESP) and control conditions (ELECT-CTL and VIS-ONLY-CTL) and both strains of mice (i.e., WT and *rd10*).

## Test Condition:

*ELECT-RESP: WT -* Latency: Both on (**S1 Col 1, Row 1**) and off (**S1 Col 2, Row 1**) latencies had a unimodal distribution. Based on the multimodality boundaries for latencies described in Carcieri *et al.* (< 400 ms for short latencies and > 400 ms for long latencies) majority of the cells had short latency response for both flashes of on and off. Duration: The duration of on response (**S1 Col 3, Row 1**) had a bimodal distribution whereas for the off responses (**S1 Col 4, Row 1**) the distribution was rather unimodal. Based on the multimodality boundaries for the duration of responses described in Carcieri *et al.* (< 200 ms for transient cells and > 200 ms for sustained cells) the on responses had both transient and sustained responses, whereas, the off responses were primarily transient. ON/OFF Index: Based on the relative amplitude of the response to flash on and flash off a bias index was calculated (see *Methods* and Carcieri *et al.* 2003). The distribution of the cells based on the bias index (termed as ON/OFF index for our study) was trimodal dividing the cells into purely on (+1), purely off (-1) and on-off (centered around 0) (**S1 Col 5, Row 1**). The cells which responded to only light onset were classified as purely on cells. The cells which responded only to light offset were classified as purely off cells. Cells which responded to both onset and offset of light were classified as on-off cells. However, based on the distribution, the number of purely on were higher in comparison to purely off cells.

*ELECT-RESP: rd10 -* Latency: Both on (**S1 Col 1, Row 2**) and off (**S1 Col 1, Row 2**) responses had latencies with multimodal distribution (primarily bimodal) with both short and long latencies. Duration: Duration of both on (**S1 Col 3, Row 2**) and off (**S1 Col 4, Row 2**) responses had a bimodal distribution containing both sustained and transient responses. ON/OFF Index: Similar to the WT retinas, based on the bias index the cells in the *rd10* retina had a trimodal distribution, classifying the cells into purely on, purely off, and on-off cells (**S1 Col 5, Row 2**). In contrast to WT retinas, the number of purely off cells were comparatively higher than purely on cells.

## Control Conditions: (i) Internal Control

*ELECT-CTL: WT -* Latency: Similar to the previous observation of the test condition (ELECT-RESP) both on and off responses had latencies with unimodal distribution, predominantly the short latency response (< 400 ms, **S1 Col 1, 2, Row 3**). Duration: Although the duration of the on responses had both transient and sustained responses, the distribution was rather unimodal with continuity from transient to sustained responses (< 200 ms transient cells, > 200 ms sustained response, **S1** **Col 3, Row 3**). However, for the duration of off responses were transient with unimodal distribution (**S1 Col 4, Row 3**). ON/OFF Index: Similar to the observation in ELECT-RESP WT cells the distribution based on the bias index was trimodal (**S1 Col 5, Row 3**), classifying the cells in purely on, purely off and purely on-off. Additionally, the number of purely off cells were substantially less in comparison to purely on cells.

*ELECT-CTL: rd10 -* Latency: Similar to the previous observation of the test condition (ELECT-RESP) both on and off responses had latencies with multimodal distribution (bimodal) with both short and long latencies (< 400 ms, short and > 400 ms long, **Fig 2. Col 1, 2, Row 4**). Duration: Duration of both on and off responses had a multimodal distribution (bimodal) with both transient and sustained cells (**S1 Col 3, 4, Row 4**). ON/OFF Index: Based on the bias index the distribution of the cells was trimodal (purely on, purely off, on-off). However, unlike the test condition (ELECT-RESP) the number of purely off cells were comparatively lower in comparison to purely on cells (**S1 Col 5, Row 4**).

## Control Conditions: (ii) External Control

*VIS-ONLY-CTL: WT -* Latency: Similar to the test and internal control condition the latency distribution for both on and off responses was unimodal with a short latency (< 400 ms, **S1 Col 1, 2, Row 5**) response. Duration: The distribution of duration of on responses were bimodal with both transient and sustained responses (**S1 Col 3, Row 5**). For the duration of off responses, the distribution was unimodal with transient responses (**S1 Col 4, Row 5**). ON/OFF Index: Similar to the observation for the test and internal control the distribution of the cells was trimodal (purely on, purely off and on-off) and the number of purely off cells were comparatively lower to purely on cells (**S1 Col 5, Row 5**).

*VIS-ONLY-CTL: rd10 -* Latency: Similar to previous observations in test and internal control condition the distribution of latencies for on and off response was bimodal with both short and long latency response (**S1 Col 1, 2, Row 6**). Duration: The distribution of duration of both on and off responses was multimodal (bimodal) with both transient and sustained responses (**S1 Col 3, 4, Row 6**). ON/OFF index: Similar to our previous observation in the internal control condition the distribution based on the bias index was trimodal (purely on, purely off, and on-off). The number of purely off cells were comparatively lower than purely on cells.

For the *rd10* retina, although the off responses had latencies with a bimodal distribution, the number of cells with short latencies (< 400 ms) were comparatively higher than the long latency responses (> 400 ms). Additionally, for the distribution of the duration of off responses, the number of transient cells (< 200 ms) was higher in comparison to sustained cells (> 200 ms).

It should be noted that for the overall distribution of response parameters described above (**S1**) we could observe a discrepancy between the number of cells for latency, duration, and the ON/OFF index. As mentioned above (see *Data Analysis*) while examining the visual response parameters (latency and duration) for flash on and flash off we excluded any response which had a peak amplitude at latencies < 100 ms (arising from sustained responses extending from an earlier phase). Additionally, responses with nonsignificant amplitude peaks were also excluded while examining these response parameters. However, while determining the ON/OFF index of the cells, relative response amplitudes from both on and off were considered. Hence all cells which had a response amplitude, either for on or off were included in the cell count. Only cells which had neither a response for light onset nor light offset were excluded from the analysis.

## Distribution of Response Parameters Based on ON/OFF Index

Next, we evaluated the distribution of visual response parameters (latency and duration) of the cells classified as on (+0.5 to 1), off (-1 to -0.5), and on-off (-0.5 to 0.5) based on the ON/OFF index (Carcieri *et al.* 2003, Sekhar *et al.* 2017). We evaluated the distribution for the test and control conditions and both strains (WT and *rd10* retina).

WT on - *Latency:* For the test condition (ELECT-RESP) and the internal control condition (ELECT-CTL) the distribution of on response latency was unimodal (**S2 Col 1, Row 1, 3**) with short latency response (< 400 ms). However, for the external control condition (VIS-ONLY-CTL) the distribution of on latency was bimodal with both short and long latency responses (> 400 ms, **S2 Col 1, Row 5**). *Duration:* For the test condition and the control conditions (both internal and external controls) the distribution of duration of on responses was multimodal (primarily bimodal, **S2 Col 1, Row 2, 4, and 6**) with both transient (< 200 ms) and sustained responses (> 200 ms).

WT off - *Latency:* For the test and the control conditions (both internal and external control conditions) the distribution of off response latency was unimodal with short latency responses (**S2 Col 2, Row 1, 3, and 5**). For the internal control condition (ELECT-CTL) we did observe few long latency responses. However, most of the cells had a distribution which was primarily unimodal. It should be noted that we did observe a cell count peak at 0. This peak corresponded to the really short latency response amplitudes (< 100 ms). To show the entire cell count distribution for off cells, we included these cells in the histogram. *Duration:* For the test condition the distribution for the duration of off responses was unimodal with transient responses (**S2 Col 2, Row 2**). However, for both the control conditions the distribution was multimodal (bimodal) with both transient and sustained responses (> 200 ms) (**S2 Col 2, Row 4, 6**).

WT on-off - *Latency:* For both the test and control conditions the distribution of on-off response latency was unimodal with short latency responses (**S2 Col 3, Row 1, 3, and 5**). *Duration:* For both test and control conditions the distribution was multimodal (bimodal) with both transient and sustained responses. (**S2 Col 3, Row 2, 4, and 6**).

*rd10* on - *Latency:* For both the test and control conditions the distribution of the latency of on responses was multimodal (bimodal) with both short and long latency responses (**S2 Col 4, Row 1, 3, and 5**). *Duration:* Similar to the latency distribution, the distribution of the duration of on responses was multimodal (primarily bimodal) with both transient and sustained responses (**S2 Col 4, Row 2, 4, and 6**). For the control conditions, some of the cells had sustained responses lasting up to 2 s.

*rd10* off - *Latency and Duration:* For both the test and control conditions the distribution was rather obscure (for both latency and duration) with most cells peaked around zero (response amplitude of latency < 100 ms). The remaining cells were distributed at various time scales and had no particular distribution. This suggests that the cells contributing to the overall distribution of off latency and off duration (**S1**) were primarily the cells with on-off responses **(S2 Col 5, Row 1-6**).

*rd10* on-off - *Latency and Duration:* For both the test and control conditions the distribution of the on-off response latency was multimodal (primarily bimodal) with both short and long latency responses. For all three conditions we did observe cells which had long latencies > 1 s and in some cells a latency up to 2 s was observed (**S2 Col 6, Row 1, 3, and 5**). Likewise, the distribution of duration of on-off responses was bimodal for both the test and control conditions with both transient and sustained responses, with few cells having a sustained response up to 2 s (**S2 Col 6, Row 2, 4, and 6**)

## Multimodality - Cell Classification

To evaluate if our data set could naturally divide into more than one class, we examined our data set for multimodality. While for the majority of visual responses in WT retina we observed a unimodal distribution, for the *rd10* retina we observed a rather multimodal distribution. This was not surprising, as the age (P28-P37) of the degenerating *rd10* mouse considered in our study had a substantial amount of viable cone photoreceptors which would contribute to the long latency, sustained responses. However, what was intriguing is that for most of the visual responses for WT retinas the responses had a shorter latency and rather a transient response. As the photoreceptors (both rods and cones) are still intact, it was surprising that we did not observe a substantial amount of long latency response. Additionally, for our data set which showed weak multimodality, the physiological response properties were rather a continuum (Carcieri *et al.* 2003, Rodieck 1998). One reason for this could be the use of full-field stimulus rather than a spot stimulus optimized to the receptive field center of each cell (see *Limitations*, below).

**S1:** **Evaluating multimodality:** Overall distribution of response parameters for on and off responses for WT and *rd10* retinas for test (ELECT-RESP) and control conditions (ELECT-CTL and VIS-ONLY-CTL). Responses measured were latency, duration, and relative response amplitudes for computing the ON/OFF index.

**S2:** **Evaluating multimodality based on ON/OFF index:** Overall distribution of latency (first row) and duration (second row) response parameters of on, off, and on-off RGC types for WT and *rd10* retinas for test (ELECT-RESP) and control conditions (ELECT-CTL and VIS-ONLY-CTL). Only on response parameters are shown for on cells, while only off parameters are shown for off cells. For on-off cells both on and off parameters are shown.

**S3-S5** Plotted as in **Figures 3-5**, but including outliers. See **S6** for cell counts of each plot.

**S6:** Cell counts for each of the box-whisker plot (for **Fig.** **3-5** and **S3-5**).

**S7:** Cell counts for on, off, on-off, and total for evaluating change in ON/OFF index, as shown in Figure 6

**S8**: Control subtracted response amplitude medians. Derived from Figure 3.
