# Supplementary figures and images for: Adaptation of visual responses in degenerating rd10 and healthy mouse retinas during ongoing electrical stimulation
Source: Front Neurosci. 2026 Mar 25;20:1730445. doi: 10.3389/fnins.2026.1730445 (PMC13022506; doi:10.3389/fnins.2026.1730445)

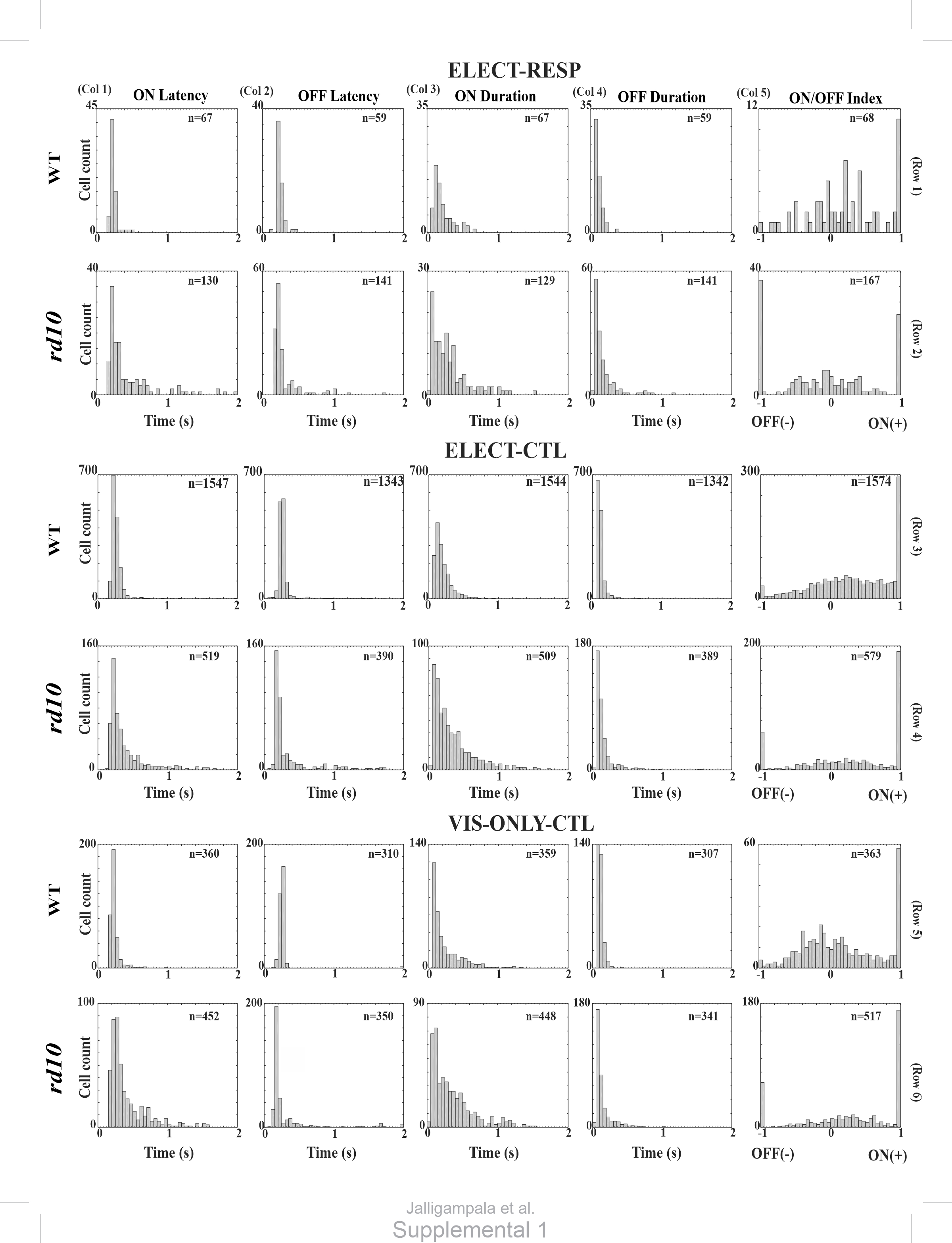

Supplement: Supplementary file 2 [file Image_1.JPEG]

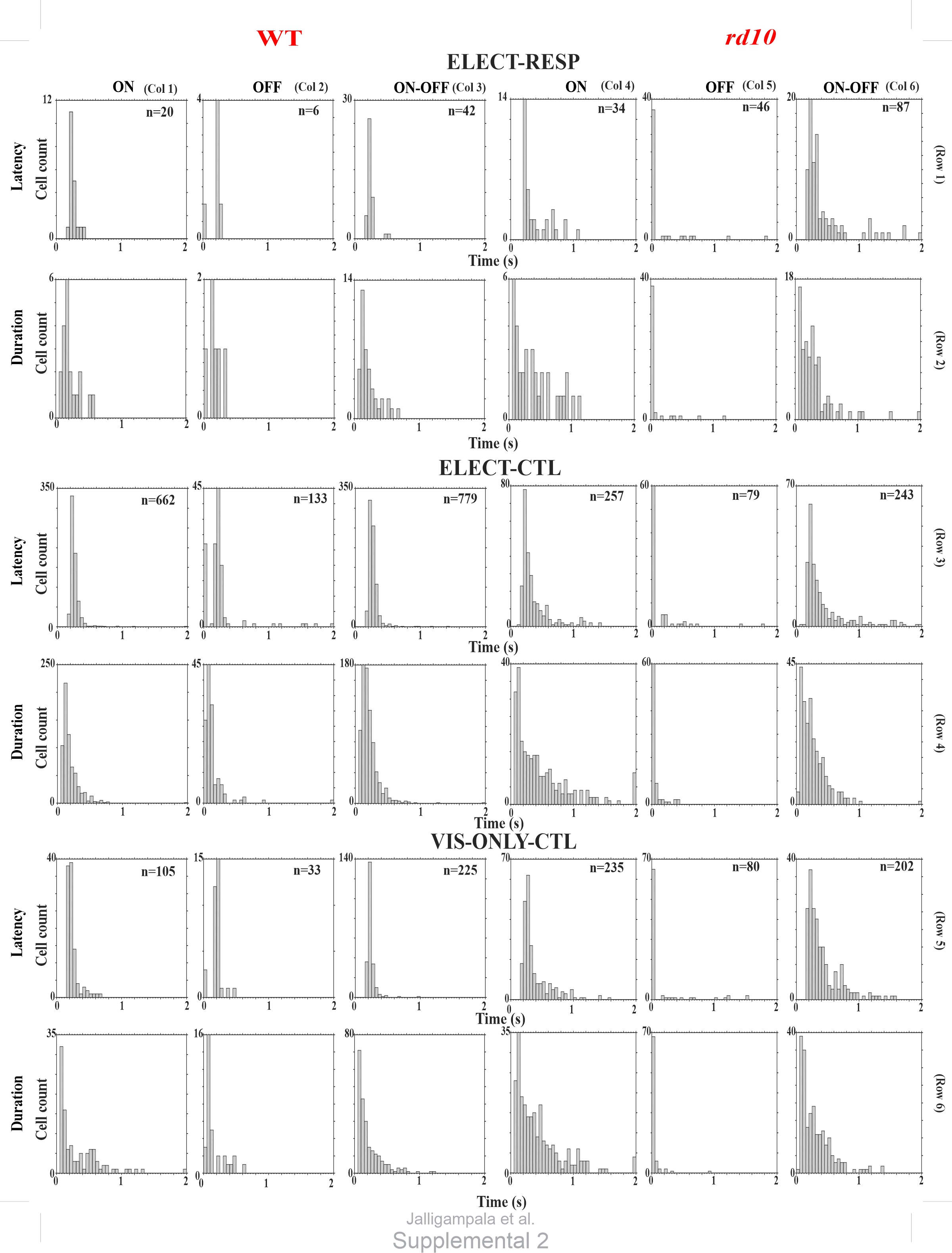

Supplement: Supplementary file 3 [file Image_2.JPEG]

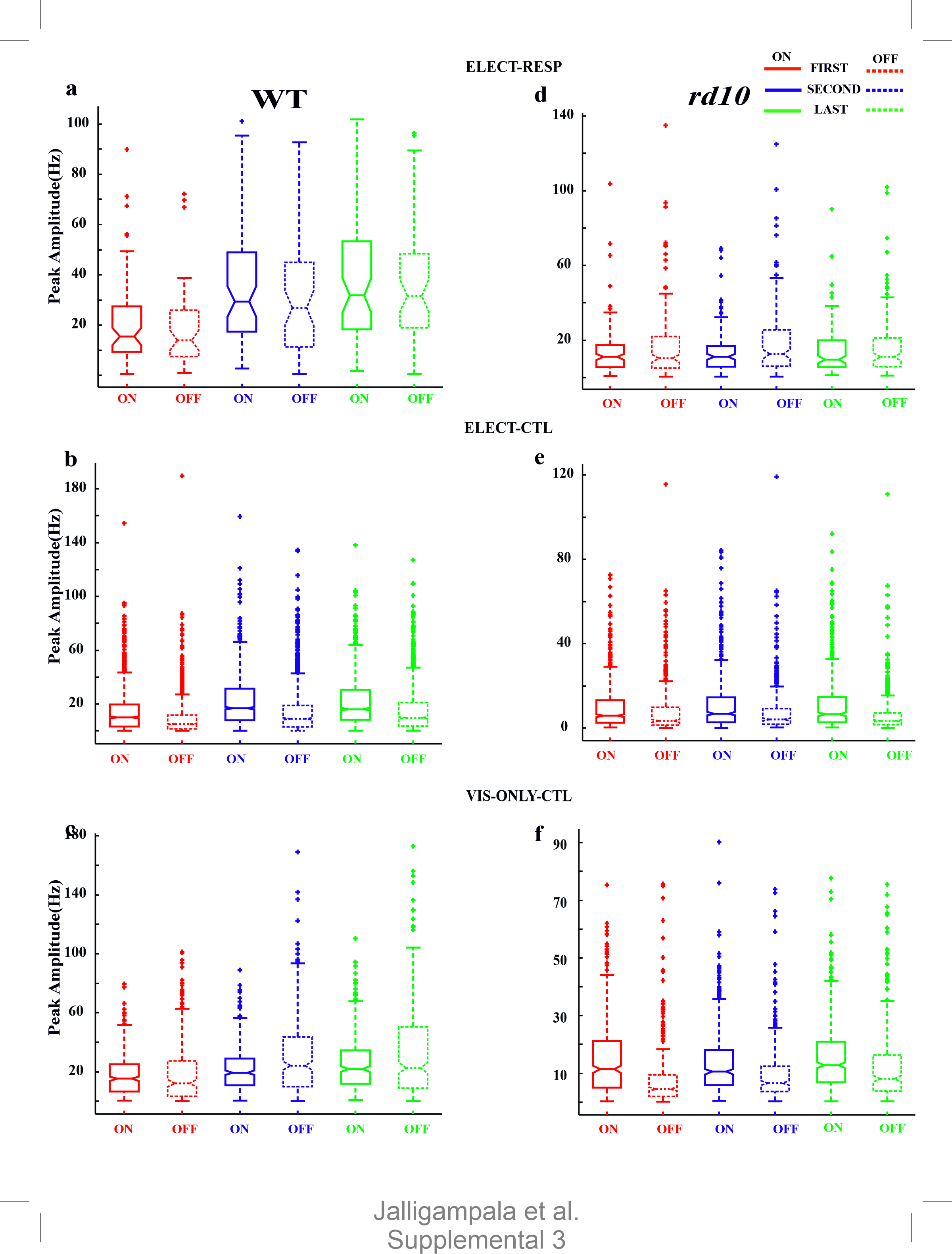

Supplement: Supplementary file 4 [file Image_3.JPEG]

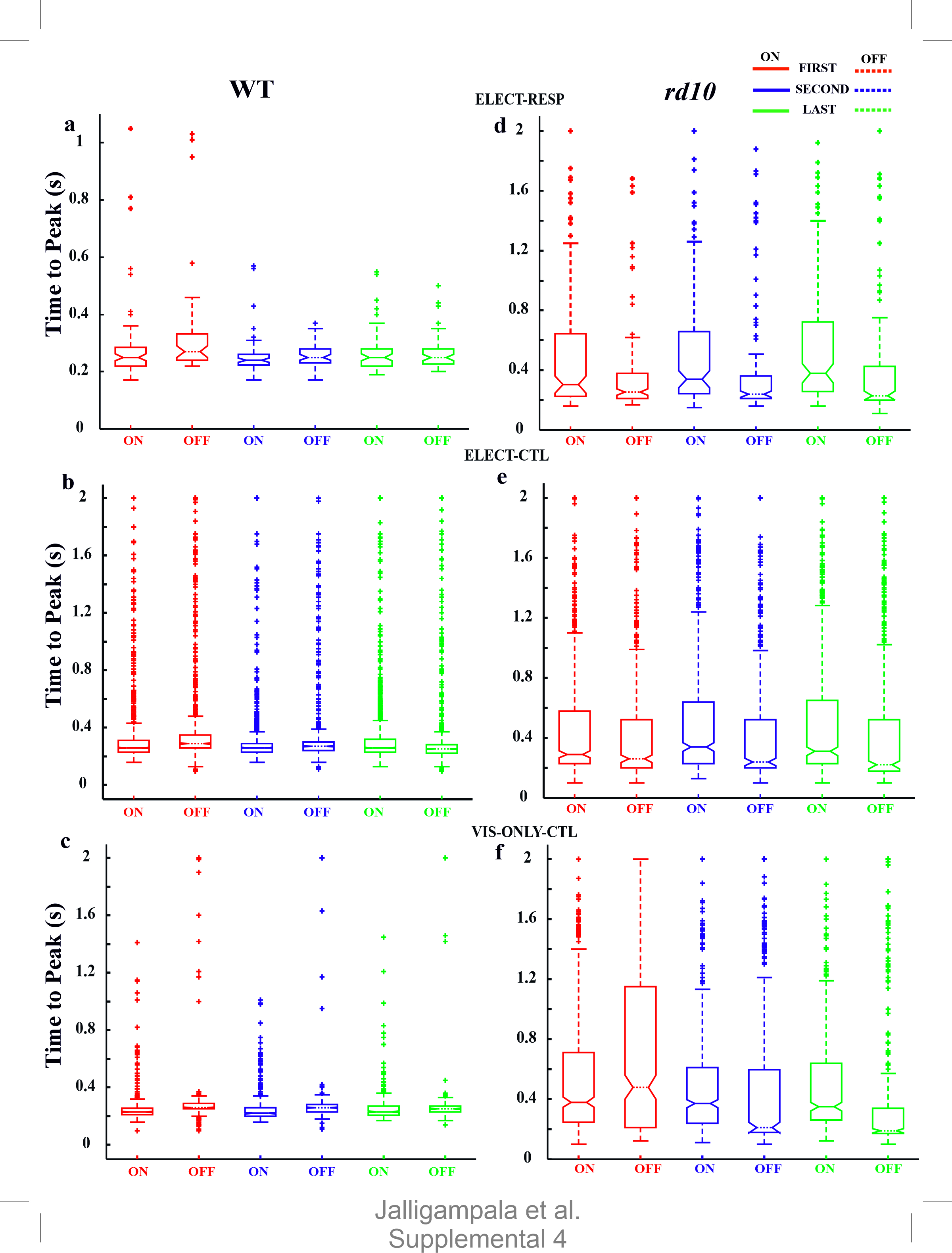

Supplement: Supplementary file 5 [file Image_4.JPEG]

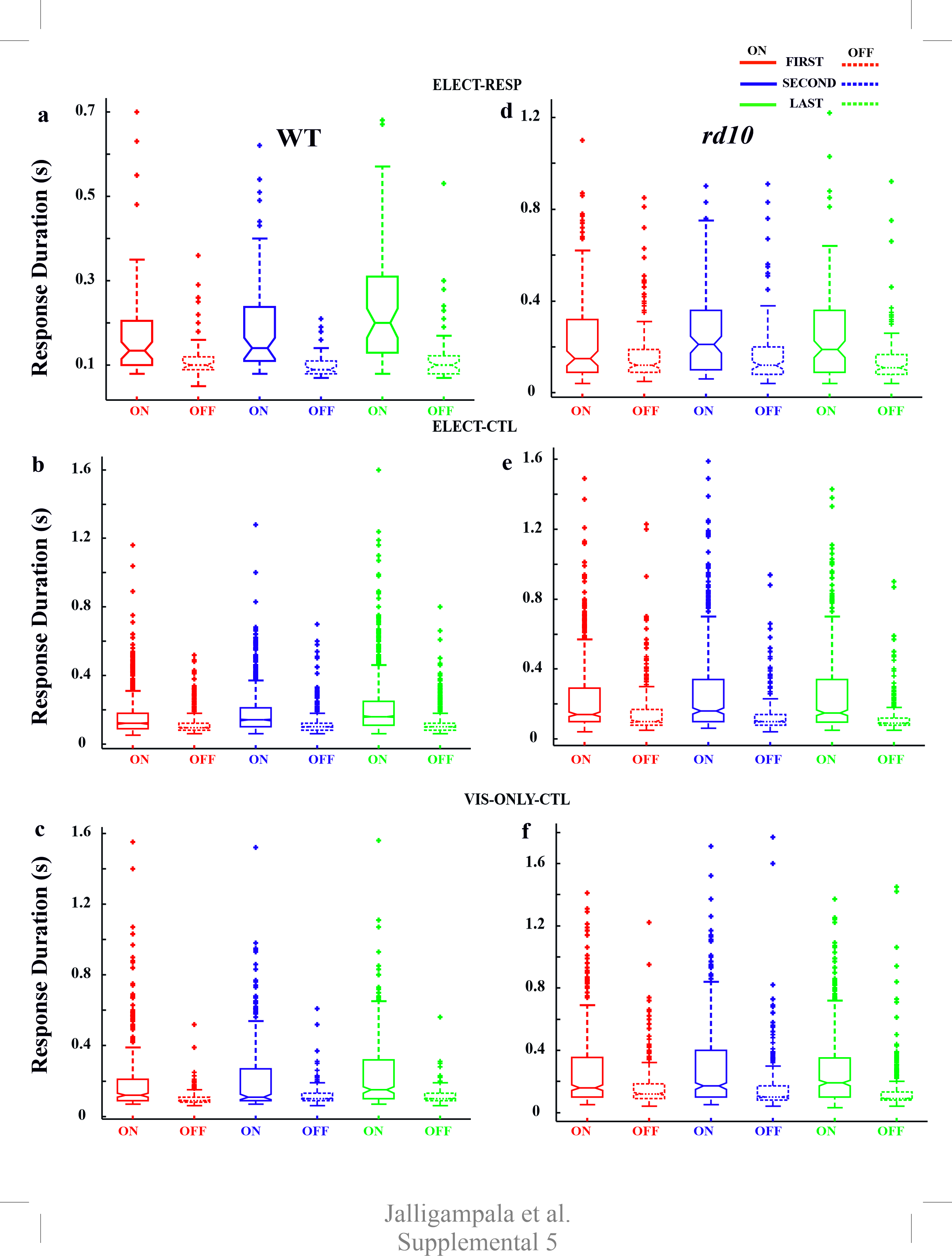

Supplement: Supplementary file 6 [file Image_5.JPEG]

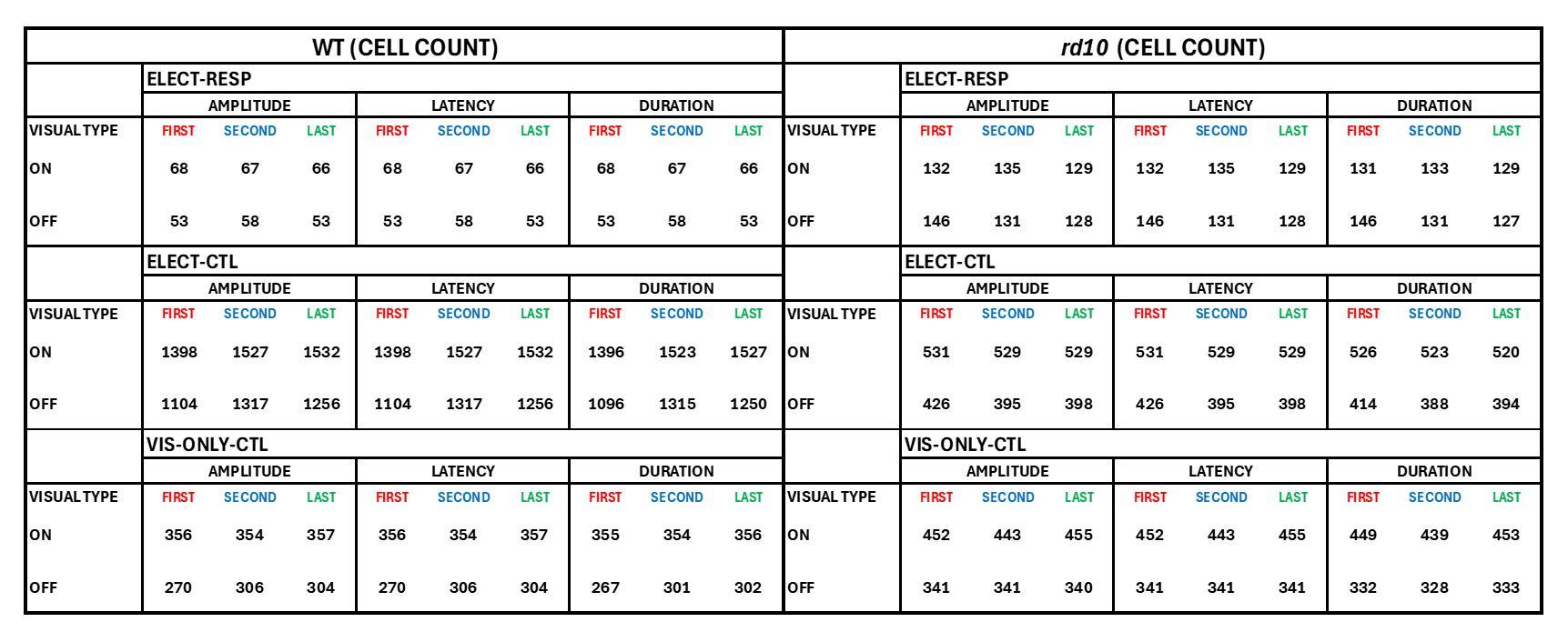

Supplement: Supplementary file 7 [file Image_6.TIF]

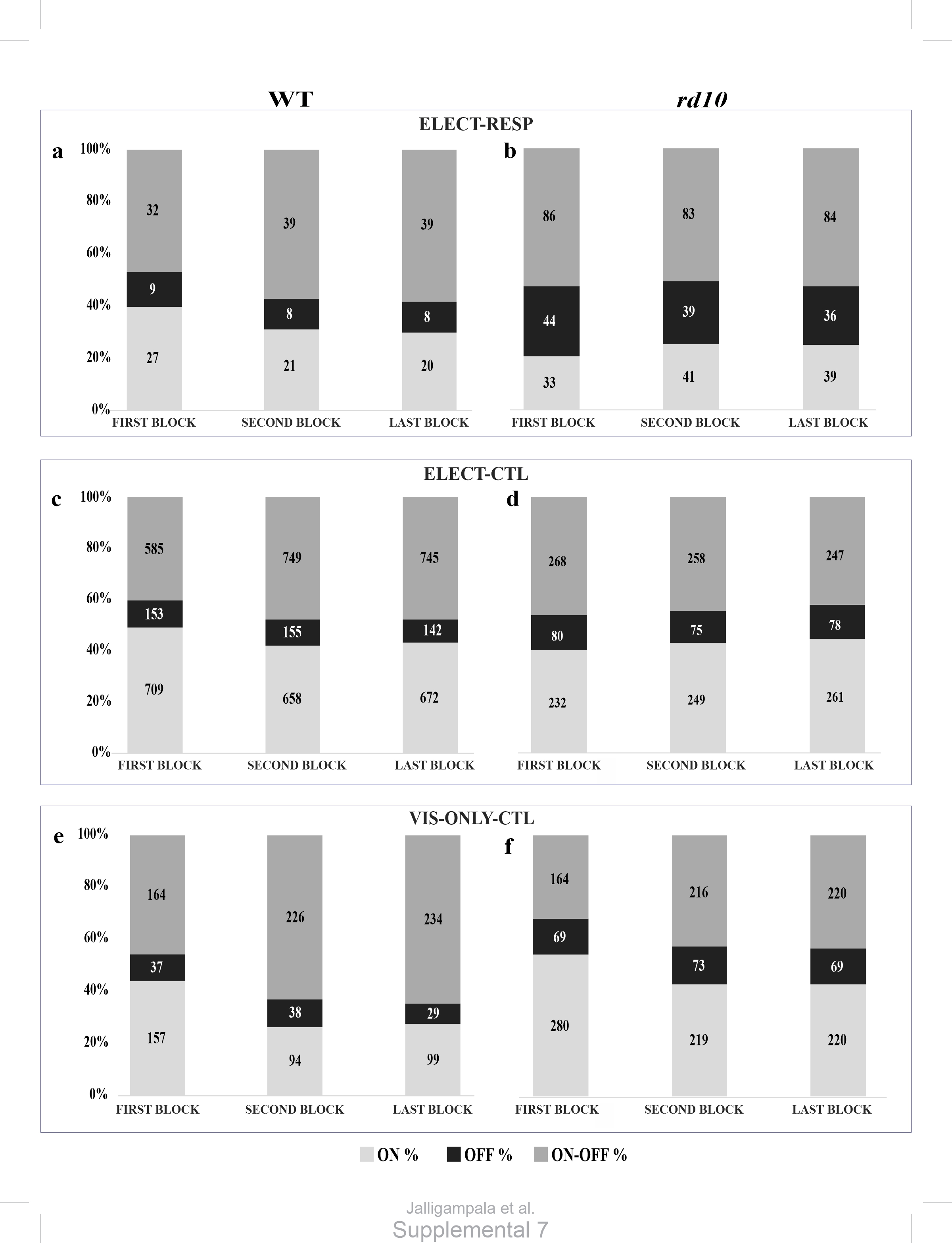

Supplement: Supplementary file 8 [file Image_7.JPEG]

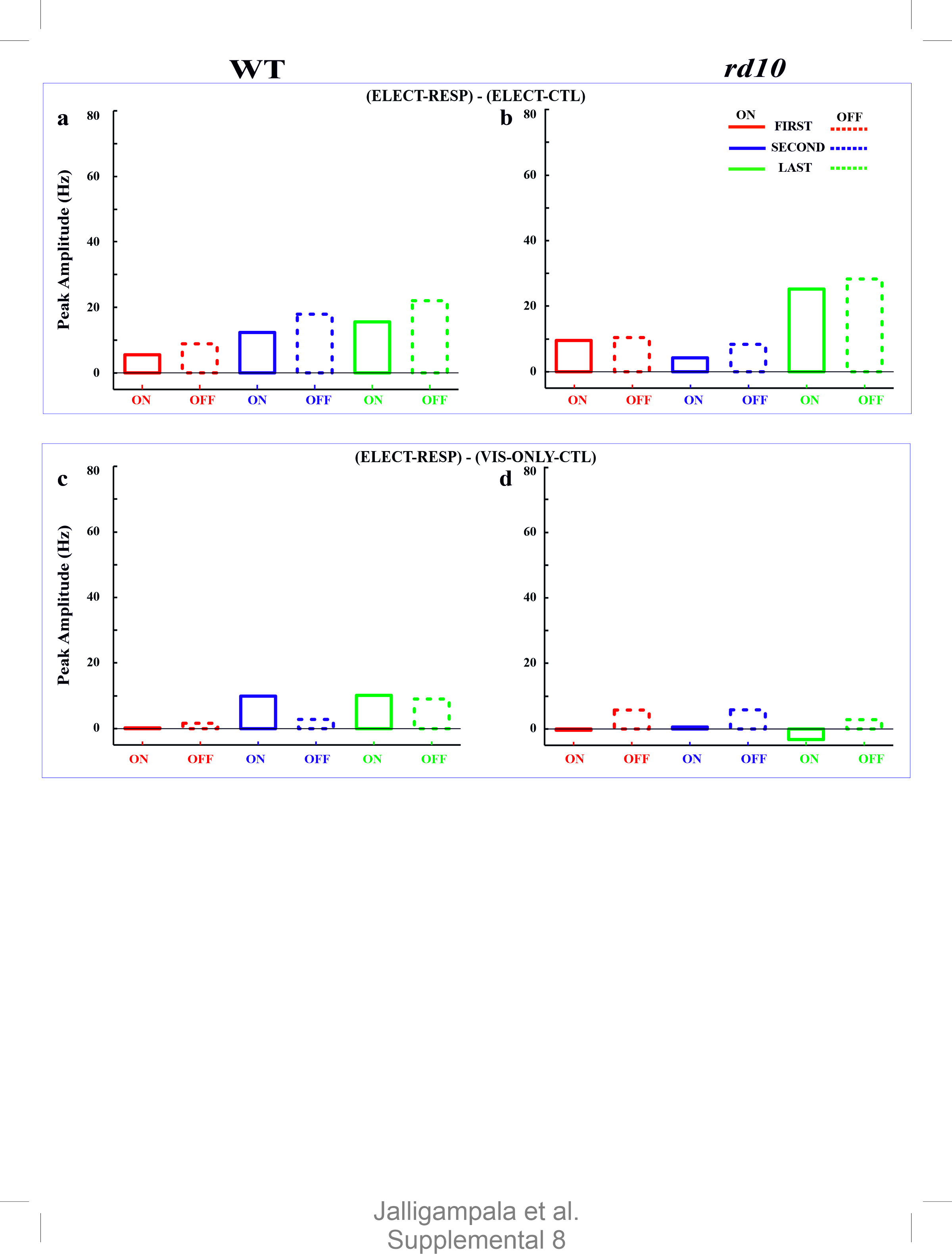

Supplement: Supplementary file 9 [file Image_8.JPEG]
